# The use of biologic adjuncts in tooth autotransplantation: a scoping review

**DOI:** 10.1007/s10006-026-01613-w

**Published:** 2026-08-07

**Authors:** Edgar Daniel Vargas-Quiroga, Brenda Carolina Pattigno-Forero, Ana Carolina Duarte Firmino, Débora S.F. Sávio, Carolina Alves Freiria de Oliveira, Isabel Schausltz Pereira Faustino, Arthur Belém Novaes

**Affiliations:** 1 Department of Oral and Maxillofacial Surgery and Periodontology, Ribeirão Preto School of Dentistry, University of São Paulo, Av. do Café, s/n, Ribeirão Preto, Ribeirão Preto, São Paulo 14040-904 Brazil; 2 Department of Pediatric Dentistry, Ribeirão Preto School of Dentistry, University of São Paulo, Ribeirão Preto, São Paulo Brazil; 3 Department of Dental Materials and Prosthodontics, Ribeirão Preto School of Dentistry, University of São Paulo, Ribeirão Preto, São Paulo Brazil; 4 Department of Stomatology, Public Oral Health and Forensic Dentistry, Ribeirão Preto School of Dentistry, University of São Paulo, Ribeirão Preto, São Paulo Brazil

**Keywords:** Transplantation, autologous, Tooth, Platelet-rich fibrin, Platelet-rich plasma, Dental enamel proteins

## Abstract

**Purpose:**

To summarize the available clinical evidence on biological adjuncts in tooth autotransplantation, including adjunct types, application protocols, procedural variables, reported outcomes, and evidence gaps.

**Methods:**

This scoping review followed JBI guidance and PRISMA-ScR. PubMed/MEDLINE, Embase, Web of Science, Scopus, and LILACS were searched from inception to 21 June 2026. Clinical human studies reporting tooth autotransplantation with at least one biologic adjunct and clinical or radiographic outcomes were included. Data were charted for study characteristics, population, autotransplanted teeth, adjunct type and application protocol, follow-up, and key findings. Critical appraisal was performed using design-specific tools.

**Results:**

Twenty-two sources published between 2005 and 2026 were included, comprising 234 patients and 257 autotransplanted teeth. Most evidence came from case reports and case series, with few comparative clinical studies. Platelet-derived preparations were the most frequently reported adjuncts, including L-PRF, A-PRF, PRP, PRGF, and CGF. Enamel matrix derivative was also reported, mainly as a root-surface application. Most studies described favorable clinical and radiographic outcomes, but protocols, root maturity, recipient-site conditions, stabilization, endodontic strategies, follow-up schedules, and outcome definitions were highly heterogeneous. Comparative and retrospective studies had serious risk of bias, mainly due to confounding.

**Conclusion:**

Biological adjuncts in tooth autotransplantation have demonstrated clinical feasibility in selected reports, but current evidence is insufficient to establish their independent benefit or superiority over standard protocols. Root maturity and procedural variables appear to be more consistent determinants of outcomes and should guide interpretation.

**Graphical Abstract:**

**Figure:**
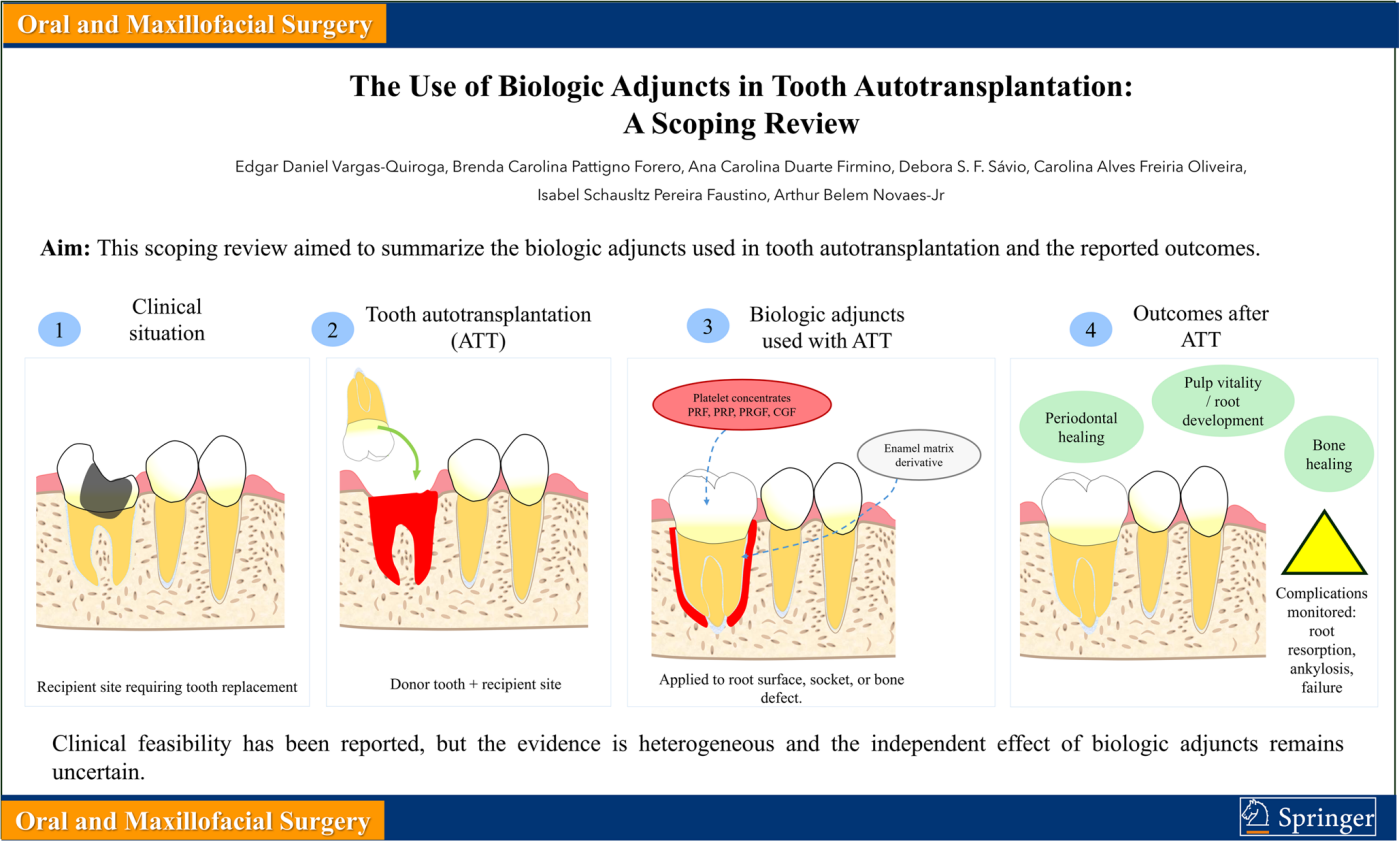

**Supplementary Information:**

The online version contains supplementary material available at 10.1007/s10006-026-01613-w.

## Introduction

Tooth autotransplantation (ATT) is a biologically based therapeutic alternative for replacing absent, lost, or unrestorable teeth within the same individual, in which a healthy tooth is transferred to another socket or to a surgically prepared recipient site [1, 2]. Its indications include teeth with an unfavorable prognosis due to extensive caries, dental trauma, developmental anomalies, and agenesis, as well as situations in which implant-supported rehabilitation should be postponed or may be contraindicated, particularly in young patients [1–4]. Beyond tooth replacement, this technique may contribute to the maintenance of the alveolar process, proprioception, and the possibility of subsequent orthodontic movement, features that reinforce its clinical relevance.

The clinical success of ATT depends primarily on preservation of the periodontal ligament (PDL), an essential structure for reinsertion of the tooth into the alveolar bone and for the maintenance of periodontal homeostasis [5, 6]. This tissue contains cells with regenerative potential, including periodontal ligament-derived stem cells, which can contribute to the repair of cementum, alveolar bone, and other supporting tissues [5]. The importance of PDL preservation has been strongly demonstrated in classical studies [7–10]. In a longitudinal series of 370 autotransplanted premolars, periodontal healing was radiographically evident in most cases after approximately 8 weeks, whereas root resorptions were usually detected within the first 6 months and were associated with the stage of root development and trauma to the PDL [7–10].

In addition to periodontal integrity, clinically relevant decisions in autotransplantation, such as the method and duration of stabilization, endodontic management, and pulpal prognosis, are closely related to the stage of root development [8, 11]. Despite the high survival and success rates reported for dental autotransplantation, complications such as inflammatory root resorption, replacement resorption, ankylosis, and persistent periapical pathology remain important clinical challenges, particularly in cases involving PDL injury, longer extra-alveolar time, or greater root maturity [12]. In this context, factors such as appropriate donor tooth selection, atraumatic extraction, proper preparation of the recipient site, and careful control of operative steps remain central to the predictability of the procedure [2, 13]. Among the prognostic factors considered most important by experienced clinicians are the stage of root development, the method used to remove the donor tooth, extraoral storage, and the condition of the root surface after removal [13].

Survival of ATT, pulpal status, root resorption, and signs of infection have also been identified as priority outcomes, reinforcing the need for greater standardization in clinical data collection and outcome reporting.

Different biological adjuncts have been investigated in ATT with the aim of supporting pulpal, periodontal, and bone healing and reducing complications such as root resorption and ankylosis [14–17]. In this review, biological adjuncts were considered biologically active or biologically derived materials applied before, during, or after ATT to modulate tissue healing. This includes enamel matrix derivatives (EMD) and autologous platelet concentrates (PCs), such as platelet-rich fibrin (PRF), platelet-rich plasma (PRP), plasma rich in growth factors (PRGF), and concentrated growth factor (CGF). Bone grafts, surgical guides, digital planning, and technical modifications were considered co-interventions when combined with a biological adjunct. These adjuncts have been proposed to support wound healing, angiogenesis, periodontal ligament repair, and soft- and hard-tissue regeneration.

This biological plausibility is supported by experimental evidence from an animal model [18], in which the addition of proliferative tissue was associated with new cementum and PDL formation, as well as reduced occurrence and extent of ankylosis and root resorption. However, the extent and nature of the clinical evidence on biological adjuncts in ATT have not been clearly characterized. It remains uncertain which adjuncts have been used clinically, how they have been incorporated into ATT protocols, and whether their reported outcomes can be distinguished from the effects of root maturity, recipient-site condition, surgical handling, stabilization, endodontic management, and co-interventions [13]. This gap is clinically relevant because biological adjuncts are often reported in complex ATT cases, but their independent contribution remains unclear. Therefore, this scoping review aimed to synthesize the clinical evidence on biological adjuncts in ATT, focusing on adjunct types, application protocols, procedural variables, reported outcomes, and evidence gaps relevant to clinical decision-making.

## Methods

### Study design

The present scoping review followed Joanna Briggs Institute (JBI) methodological guidance and adhered to the Preferred Reporting Items for Systematic Reviews and Meta‑Analyses Extension for Scoping Reviews (PRISMA‑ScR) [19].

The review question was structured according to the PCC model (Participants, Concept, Context), considering human patients undergoing ATT, the use of biological adjuncts and the associated outcomes, and the clinical context in which autotransplantation procedures are performed. The review protocol was registered in the Open Science Framework (OSF): 10.17605/OSF.IO/DF6XB.

### Literature search and search strategy

A comprehensive literature search was conducted in MEDLINE via PubMed, Embase, Web of Science, Scopus, and LILACS via the Virtual Health Library, from database inception to 21 June 2026, with no restriction on year of publication. No language filters were applied at the search stage.

The search strategies were structured around two domains: ATT and biological adjuncts. These domains were combined as ATT-related terms AND biological adjunct-related terms. Within each domain, synonyms and related terms were combined using OR. Controlled vocabulary was combined with free-text terms when available. The ATT domain included terms for tooth/dental autotransplantation, autogenous tooth transplantation, autologous tooth transplantation, and related variants. The biological adjunct domain included terms for PCs, including PRF, PRP, PRGF, and CGF, as well as EMD, hyaluronic acid (HA), and recombinant growth factors (RGFs), such as platelet-derived growth factor (PDGF). A summary of the search strategy domains and the main terms used is presented in Table 1. ClinicalTrials.gov was searched on 28 June 2026 as a complementary registry source. The complete conceptual search blocks, database-specific strategies, and complementary ClinicalTrials.gov search are provided in Online Resource 1, Sect. 2.

**Table 1:** Summary of electronic database searches and records retrieved

Database	Search approach	Main search structure	Records retrieved
MEDLINE via PubMed	MeSH terms combined with free-text terms	Tooth autotransplantation terms AND biological adjunct terms	166
Embase	Emtree terms combined with title, abstract, and keyword terms	Tooth transplantation/autotransplantation terms AND biological adjunct terms	216
Web of Science	All-fields free-text search	Tooth autotransplantation terms AND biological adjunct terms	264
Scopus	TITLE-ABS-KEY search using free-text terms and proximity operators	Tooth autotransplantation terms within 25 words of biological adjunct terms	93
LILACS via Virtual Health Library	Free-text terms in English, Portuguese, and Spanish	Tooth autotransplantation terms AND biological adjunct terms across three languages	114
Total	—	—	853

*Emtree* Embase subject headings, *MeSH* Medical Subject Headings

### Eligibility criteria

For this review, a biological adjunct was defined as a biologically active or biologically derived material used before, during, or after ATT to support periodontal, pulpal, soft-tissue, or bone healing. This included PCs, EMD, recombinant growth factors, hyaluronic acid, and similar products. Bone grafts, surgical guides, tooth replicas, digital planning, and other technical or surgical procedures were not considered biological adjuncts and, when used alongside one, were recorded as co-interventions.

Studies were selected based on predefined inclusion and exclusion criteria. Titles and abstracts of retrieved records were screened against the eligibility criteria, and potentially relevant articles were retrieved in full text for detailed assessment.

Studies were included if they: (1) enrolled human patients undergoing tooth autotransplantation; (2) reported the use of at least one biological adjunct applied at any stage of the autotransplantation procedure, including preoperative, intraoperative, or postoperative application; (3) reported clinical and/or radiographic outcomes related to the transplanted tooth or surrounding tissues, such as survival or success, periodontal status, pulp status, root resorption, ankylosis, mobility, or complications; (4) reported a follow-up period longer than 6 months; (5) were clinical studies in humans, including case reports, case series, observational studies, or randomized or non-randomized clinical trials; and (6) had full text available in English, Portuguese, or Spanish.

Studies were excluded if they: (1) were in vitro experiments or animal studies; (2) focused exclusively on tooth replantation, intentional replantation, allotransplantation, or xenotransplantation; (3) were non-original articles, including narrative or systematic reviews, editorials, letters, or conference abstracts without full data; (4) were registry entries, clinical trial protocols, preprints, theses, or unpublished reports without published clinical outcome data; (5) reported a follow-up period of 6 months or less; (6) did not clearly identify the biological adjunct or did not relate it to the autotransplant procedure; or (7) did not report extractable clinical or radiographic outcomes.

Studies with mixed samples were considered eligible only when tooth autotransplantation cases involving biological adjuncts could be identified. When outcomes were not fully stratified by procedure type, findings were extracted when possible and interpreted with caution.

### Study selection

All search results were exported and imported into Rayyan (Rayyan Systems Inc., Qatar) for citation management and screening. Rayyan’s automatic duplicate detection was complemented by manual checking before screening proceeded. Two independent reviewers, E.D.V.Q. and B.C.P.F., screened titles and abstracts according to the eligibility criteria, excluding clearly ineligible records. Potentially relevant records were retained for full-text assessment. The same reviewers independently assessed the full texts to confirm eligibility. Disagreements were resolved by consensus and, when necessary, by a third reviewer, A.C.D.F.

### Data extraction

Data were extracted into a standardized Microsoft Excel spreadsheet (Microsoft 365) by E.D.V.Q., and B.C.P.F., with verification by A.C.D.F. and D.S.F.S. The following variables were charted from each included source: author and year, country, study design, sample size, population characteristics, transplant characteristics, biological adjunct and application protocol, follow-up duration, and key findings. Extracted data were checked for consistency and are summarized in Table 2.

**Table 2:** Summary of study characteristics and clinical categorization

Domain	Category	Included sources	Summary
Study design	Case reports	Chaudhary et al.; Miura et al.; Alkofahi et al.; Gaviño-Orduña et al.; Mena-Álvarez et al.; Rey Lescure et al.; Sakhariya et al.; Alijani et al.; Adamska et al.; Shetty et al.; Ainiwaer and Wang; SamavatiJame et al.; Ma et al. [21–33]	Case reports represented the largest proportion of the evidence and mainly described technical feasibility, adjunct application, and individual clinical outcomes.
Study design	Case series	Hamamoto et al.; Gonzalez-Ocasio and Stevens; Anitua et al.; Pedrinaci et al.; Ainiwaer et al. [14, 20, 34–36]	Case series provided broader clinical descriptions but remained heterogeneous in sample size, follow-up, adjunct type, and outcome reporting.
Study design	Comparative or retrospective studies	Marques-Ferreira et al.; Keranmu et al.; Genc et al.; Ronchetti et al. [15–17, 37]	These studies provided comparative or larger retrospective data, but interpretation was limited by methodological heterogeneity and risk of bias.
Root maturity	Open apex/immature teeth	Gonzalez-Ocasio and Stevens; Alkofahi et al.; Rey Lescure et al.; Adamska et al.; SamavatiJame et al.; Ma et al.; Anitua et al. [23, 26, 29, 31, 32, 34, 35]	Immature teeth were generally managed without immediate endodontic treatment, with monitoring for pulp sensibility, continued root development, and apical closure.
Root maturity	Closed apex/mature teeth	Hamamoto et al.; Keranmu et al.; Miura et al.; Gaviño-Orduña et al.; Mena-Álvarez et al.; Alijani et al.; Ainiwaer and Wang; Pedrinaci et al.; Ainiwaer et al. [14, 16, 20, 22, 24, 25, 28, 30, 36]	Mature teeth were more commonly managed with planned, early, or selective RCT.
Root maturity	Mixed or unclear root maturity	Marques-Ferreira et al.; Ronchetti et al.; Genc et al.; Chaudhary et al.; Sakhariya et al.; Shetty et al. [15, 17, 21, 27, 33, 37]	Some studies included mixed root development stages or did not clearly report root maturity, limiting direct comparison across protocols.
Recipient-site status	Fresh extraction sockets	Gonzalez-Ocasio and Stevens; Alkofahi et al.; Rey Lescure et al.; Alijani et al.; SamavatiJame et al. [23, 26, 28, 31, 34]	Fresh extraction sockets were commonly used in reports involving immature teeth or immediate replacement of non-restorable teeth.
Recipient-site status	Surgically or digitally prepared sockets	Gaviño-Orduña et al.; Mena-Álvarez et al.; Shetty et al.; Ma et al.; Pedrinaci et al.; Ainiwaer and Wang [20, 24, 25, 30, 32, 33]	Several studies used socket preparation, digital planning, surgical guides, tooth replicas, or physio-dispenser-based preparation.
Recipient-site status	Defective, infected, or reconstructed sites	Hamamoto et al.; Chaudhary et al.; Miura et al.; Keranmu et al.; Anitua et al.; Sakhariya et al.; Adamska et al.; Ainiwaer et al.; Ainiwaer and Wang [14, 16, 21, 22, 27, 29, 30, 35, 36]	Defective, infected, or reconstructed recipient sites frequently required adjunctive grafting, membrane use, or additional regenerative procedures.

*ATT* autotransplantation, *CGF* concentrated growth factor, *EMD* enamel matrix derivative, *L-PRF* leukocyte- and platelet-rich fibrin, *NR* not reported, *PRF* platelet-rich fibrin, *PRGF* plasma rich in growth factors, *PRP* platelet-rich plasma, *RCT* root canal treatment

### Critical appraisal

Critical appraisal was performed independently by C.A.F.O. using design-specific tools. Case reports were assessed with the JBI Critical Appraisal Checklist for Case Reports, case series with the JBI Critical Appraisal Checklist for Case Series, and comparative or retrospective clinical studies with Risk Of Bias In Non-randomized Studies of Interventions tool (ROBINS-I). Disagreements were resolved through discussion and consensus, with adjudication by I.S.P.F. when necessary. No study was excluded based on critical appraisal results, which were used to contextualize the methodological rigor and limitations of the mapped evidence. Results are presented in Online Resource1, Tables S6-S8.

## Results

### Study selection

A total of 853 records were identified through database searches in MEDLINE via PubMed, Embase, Web of Science, Scopus, and LILACS via the Virtual Health Library. After duplicate removal (*n* = 360), 493 unique records were screened by title and abstract. During this stage, the two reviewers reached the same decision for 484 of 493 records, corresponding to a percentage agreement of 98.2%. Cohen’s kappa was 0.90, indicating almost perfect agreement. The nine disagreements were resolved through discussion and, when necessary, adjudication by a third reviewer.

**Fig. 1:**
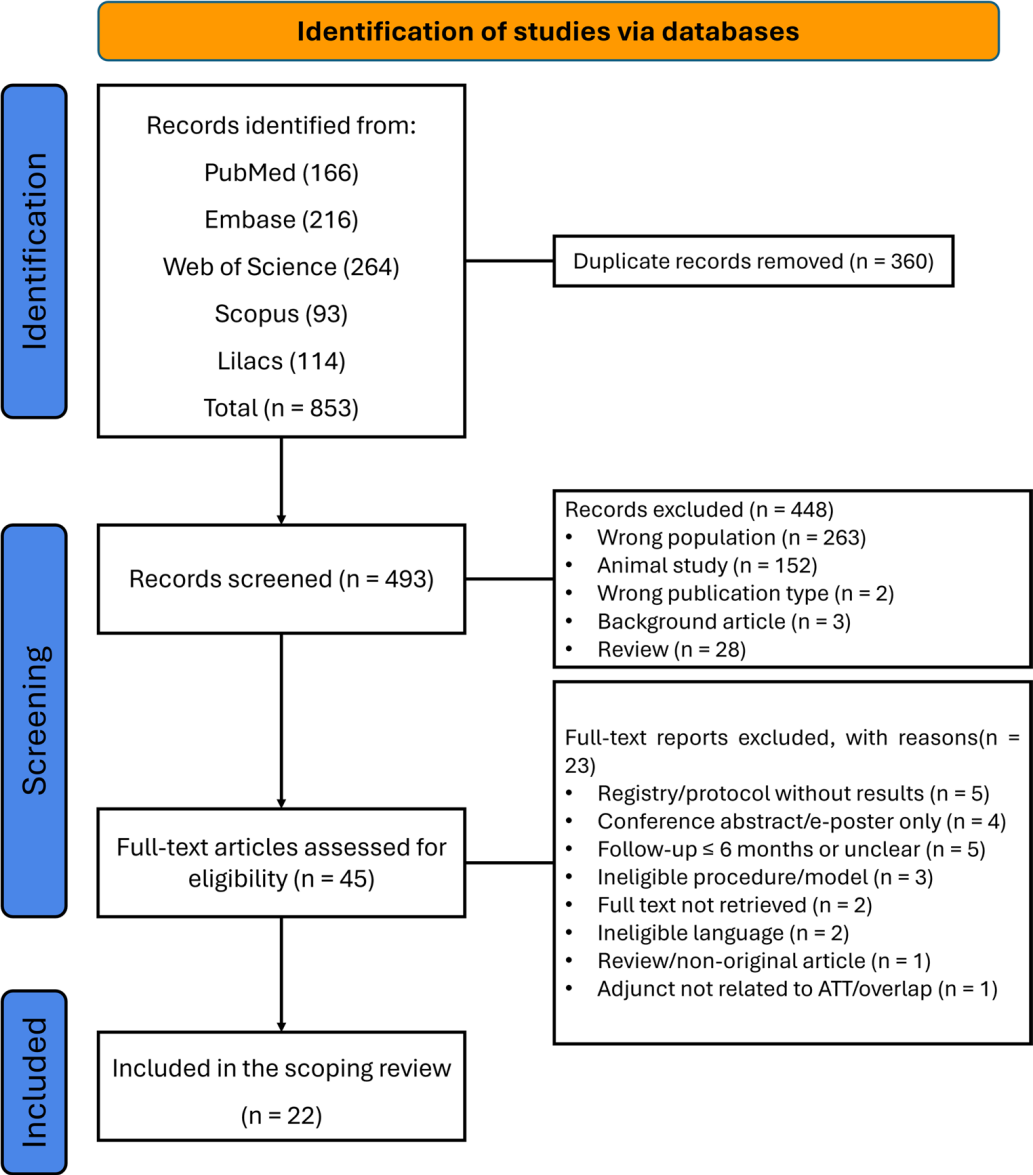
PRISMA flowchart

After conflict resolution, 448 records were excluded, and 45 reports were retrieved for full-text assessment. After full-text evaluation, 23 reports were excluded for predefined reasons, which are detailed in Online Resource 1, Supplementary Table S1. Therefore, 22 sources of evidence were ultimately included in this scoping review (Fig. 1). The complementary ClinicalTrials.gov search retrieved 12 records with the broad ATT-related strategy and 6 records with the adjunct-specific strategy. No additional source of evidence was identified. Registry records without published clinical outcome data were excluded, and the registry record corresponding to the included study by Pedrinaci et al. [20] was treated as a related registry entry.

### Characteristics of included sources

The 22 included sources were published between 2005 and 2026. The evidence base consisted of 13 case reports [21–33], five case series, including prospective and retrospective designs [14, 20, 34–36], three comparative clinical studies [15–17], and one retrospective analysis [37]. Across all included sources, 234 patients were described. In total, 257 transplanted teeth were reported. The included sources were geographically distributed across 12 countries. China and Spain contributed the highest number of studies, with four studies each, followed by India, with three studies, and Japan and Switzerland, with two studies each. The remaining studies were conducted in the United States, Iran, Jordan, Libya, Poland, Portugal, and Türkiye, with one study each.

The main characteristics and clinical categories of the included sources are summarized in Table 2, and detailed study-level characteristics are provided in Online Resource 1, Supplementary Table S2. Donor teeth were most commonly posterior teeth, particularly third molars [16, 20, 23, 25–27, 29–32, 34, 36]. Other donor teeth included premolars, canines, supernumerary teeth, and maxillary molars [14, 15, 22, 24, 28, 33]. Recipient sites were frequently first or second molar regions requiring replacement due to extensive caries, endodontic failure, periapical pathology, or structural compromise [15–17, 20, 23, 27, 30, 31, 34, 36, 37]. Recipient-site conditions were heterogeneous and included fresh extraction sockets, surgically prepared sockets, defective sockets, sites with chronic periapical lesions, and areas requiring bone reconstruction.

Root development stage also varied across studies. Immature donor teeth with open apices were reported [23, 26, 29, 31, 32, 34, 35]. Mature donor teeth with complete root formation or closed apices were reported [14, 16, 20, 24, 25, 28, 30, 36]. Marques-Ferreira et al. [15], Ronchetti et al. [37], and Genc et al. [17] included mixed root development stages. In other reports, root maturity was not clearly stated or was only indirectly inferred from the clinical description [21, 27, 33]. One study included a mixed sample of conventional ATT and intentional replantations [15].

### Biological adjuncts and application patterns

The biological adjuncts and procedural protocols are summarized in Table 3, and detailed study-level protocol data are provided in Online Resource 1, Supplementary Tables S3 and S4. Platelet-derived preparations were the most frequently reported category. PRF-based products, including leukocyte- and platelet-rich fibrin (L-PRF) and advanced platelet-rich fibrin (A-PRF), were reported in several studies [21, 23, 26–29, 31–33]. PRP was reported in four studies [17, 22, 27, 34], PRGF in three studies [24, 25, 35], and CGF in three studies [16, 30, 36]. EMD was reported in four studies [14, 15, 20, 37].

**Table 3:** Summary of biological adjuncts and procedural protocols

Procedural domain	Category	Included sources	Main protocol pattern	Relevant considerations
Biological adjunct	EMD	Hamamoto et al.; Marques-Ferreira et al.; Pedrinaci et al.; Ronchetti et al. [14, 15, 20, 37]	EMD was generally applied directly to the donor root surface before transplantation when the application protocol was reported.	Used mainly in mature or mixed-root populations; comparative evidence did not clearly isolate an independent EMD effect.
Biological adjunct	PRF-based products	Chaudhary et al.; Alkofahi et al.; Rey Lescure et al.; Alijani et al.; Adamska et al.; Shetty et al.; SamavatiJame et al.; Ma et al. [21, 23, 26, 28, 29, 31–33]	PRF, L-PRF, or A-PRF were commonly placed in the recipient socket, around the transplanted root, or within peri-radicular or bone defects.	Frequently combined with grafting materials or digital/surgical co-interventions, limiting interpretation of the adjunct alone.
Biological adjunct	PRP	Miura et al.; Sakhariya et al.; Gonzalez-Ocasio and Stevens; Genc et al. [17, 22, 27, 34]	PRP was generally injected or packed into the recipient site, alone or mixed with grafting material.	Genc et al. provided comparative data but did not show a clear short-term benefit of PRP over controls.
Biological adjunct	PRGF	Gaviño-Orduña et al.; Mena-Álvarez et al.; Anitua et al. [24, 25, 35]	PRGF was used as a liquid, clot, membrane, or storage medium during the extraoral phase and/or recipient-site preparation.	Often associated with digital planning, 3D replicas, or bone-graft procedures.
Biological adjunct	CGF	Keranmu et al.; Ainiwaer and Wang; Ainiwaer et al. [16, 30, 36]	CGF was mainly applied as membranes placed in sockets, periapical defects, or grafted recipient areas.	Mostly used in infected, defective, or reconstructed recipient sites.
Co-interventions	Grafting materials or membranes	Chaudhary et al.; Miura et al.; Sakhariya et al.; Adamska et al.; Anitua et al.; Shetty et al.; Ainiwaer et al. [21, 22, 27, 29, 33, 35, 36]	Adjuncts were frequently combined with DFDBA, PCBM, autogenous bone, autogenous dentin graft, artificial bone powder, or membranes.	These co-interventions make it difficult to isolate the effect of the biological adjunct.
Co-interventions	Digital planning, guides, or replicas	Gaviño-Orduña et al.; Mena-Álvarez et al.; Pedrinaci et al.; Ainiwaer and Wang; Ma et al. [20, 24, 25, 30, 32]	Digital workflows included CBCT/IOS planning, 3D replicas, surgical guides, or seating guides.	Technical precision and reduced extraoral time may have influenced outcomes independently of the biological adjunct.
Stabilization	Short-term non-rigid or semi-rigid stabilization	Hamamoto et al.; Marques-Ferreira et al.; Keranmu et al.; Genc et al.; Chaudhary et al.; Mena-Álvarez et al.; Rey Lescure et al.; Alijani et al.; Ainiwaer and Wang; Gonzalez-Ocasio and Stevens; Pedrinaci et al.; Ainiwaer et al.; Shetty et al. [14–17, 20, 21, 25, 26, 28, 30, 33, 34, 36]	Most reported stabilization protocols lasted 1 to 4 weeks and used sutures, wire-and-resin splints, orthodontic wire, archwire-based stabilization, or fiberglass bands.	Wire diameter was rarely reported; when available, 24G stainless-steel wire and 0.016-inch stainless-steel wire were described.
Stabilization	Longer stabilization	Adamska et al.; Ma et al. [29, 32]	Longer stabilization periods of approximately 2 to 3 months were reported in a limited number of studies.	Longer stabilization may reflect surgical complexity, recipient-site condition, or stability concerns.
Endodontic strategy	Planned or early RCT	Hamamoto et al.; Keranmu et al.; Mena-Álvarez et al.; Sakhariya et al.; Alijani et al.; Ainiwaer and Wang; Shetty et al.; Pedrinaci et al.; Ainiwaer et al. [14, 16, 20, 25, 27, 28, 30, 33, 36]	Mature teeth were frequently treated with planned, early, or prophylactic RCT, often within the first postoperative month.	RCT protocols varied in timing and reporting detail.
Endodontic strategy	No immediate RCT/monitoring	Gonzalez-Ocasio and Stevens; Alkofahi et al.; Rey Lescure et al.; Adamska et al.; SamavatiJame et al.; Ma et al.; Anitua et al. [23, 26, 29, 31, 32, 34, 35]	Immature teeth were generally monitored clinically and radiographically for pulp vitality or sensibility, root development, and apical closure.	No included study clearly described regenerative endodontic therapy as part of the ATT protocol.
Endodontic strategy	Selective or delayed RCT	Marques-Ferreira et al.; Genc et al.; Ronchetti et al. [15, 17, 37]	RCT was performed when signs of necrosis, periapical pathology, or other unfavorable changes were detected.	Selective treatment reflects clinical decision-making but complicates comparison across studies.

*A-PRF* advanced platelet-rich fibrin, *ATT* autotransplantation, *CGF* concentrated growth factor, *DFDBA* demineralized freeze-dried bone allograft, *EMD* enamel matrix derivative, *L-PRF* leukocyte- and platelet-rich fibrin, *NR* not reported, *PCBM* particulate cancellous bone and marrow, *PRF* platelet-rich fibrin, *PRGF* plasma rich in growth factors, *PRP* platelet-rich plasma, *RCT* root canal treatment

Adjunct application strategies were heterogeneous. EMD was generally applied directly to the donor root surface before transplantation [14, 15, 20]. PRF-based products were commonly used as clots or membranes placed in the recipient socket, around the transplanted root, or within peri-radicular or bone defects [21, 23, 26, 28, 29, 31–33]. PRP and PRGF were generally used as liquid or clot preparations [22, 24, 25, 27, 34, 35], whereas CGF was mainly applied as membranes in sockets or defects [16, 30, 36].

Several studies combined biological adjuncts with other interventions. These included demineralized freeze-dried bone allograft in Chaudhary et al. [21], particulate cancellous bone and marrow in Miura et al. [22], autogenous bone in Sakhariya et al. [27] and Anitua et al. [35], autogenous dentin graft in Adamska et al. [29], artificial bone powder in Ainiwaer et al. [36], and allogenic bone graft with a bioresorbable membrane in one case reported by Shetty et al. [33]. Digital planning, surgical guides, or tooth replicas were reported by Gaviño-Orduña et al. [24], Mena-Álvarez et al. [25], Pedrinaci et al. [20], Ainiwaer and Wang [30], and Ma et al. [32].

### Stabilization and endodontic strategies

Stabilization methods varied across studies but were generally short-term and based on non-rigid or semi-rigid approaches. When reported, splinting was most maintained for 1 to 4 weeks using sutures, wire-and-resin splints, orthodontic wire with composite resin, archwire-based stabilization, or fiberglass bands [14–17, 20, 21, 25, 26, 28, 30, 33, 34, 36]. Longer stabilization periods, up to 2 or 3 months, were less frequent and were reported by Adamska et al. [29] and Ma et al. [32].

Details on splint rigidity were inconsistently reported. In most studies, the wire diameter was not specified. Only a limited number of reports provided this information, including the use of 24G stainless-steel wire by Sakhariya et al. [27] and 0.016-inch stainless-steel wire by Adamska et al. [29].

Endodontic management differed according to root development stage and study protocol. Teeth with complete root formation were more frequently managed with planned or early root canal treatment (RCT), usually within the first postoperative month [14, 16, 20, 25, 27, 28, 30, 33, 36]. In contrast, immature teeth with open apices were generally managed without immediate endodontic intervention, with follow-up focused on pulp vitality or sensibility, continued root development, and apical closure [23, 26, 29, 31, 32, 34, 35].

No included study clearly described regenerative endodontic therapy as part of the autotransplantation protocol for immature teeth. In these cases, the usual approach was clinical and radiographic monitoring, with endodontic treatment reserved for signs of pulp necrosis, infection, periapical pathology, or progressive inflammatory resorption. In mixed populations, Marques-Ferreira et al. [15] reported a greater need for RCT in teeth with complete root formation than in teeth with incomplete root formation, whereas Genc et al. [17] adopted a selective approach without routine prophylactic endodontic treatment.

### Follow-up intervals, outcomes, and complications

Follow-up intervals, outcome domains, and complications are summarized in Table 4, and detailed study-level outcome data are provided in Online Resource 1, Supplementary Table S5. Follow-up duration ranged from 12 months to 12 years [17, 21, 29, 34, 37]. Short- and medium-term follow-up, approximately 12 to 36 months, predominated. Longer-term outcomes were available only in a limited number of sources, mainly case reports, case series, and retrospective analyses [31, 35–37].

**Table 4:** Summary of follow-up, reported outcome domains, and complications

Outcome domain	Main context/studies	Summary of findings	Main complications or limitations
Follow-up intervals	All included sources: Hamamoto et al.; Marques-Ferreira et al.; Chaudhary et al.; Miura et al.; Ronchetti et al.; Gonzalez-Ocasio and Stevens; Alkofahi et al.; Gaviño-Orduña et al.; Mena-Álvarez et al.; Keranmu et al.; Rey Lescure et al.; Anitua et al.; Sakhariya et al.; Alijani et al.; Adamska et al.; Pedrinaci et al.; Shetty et al.; Ainiwaer et al.; Ainiwaer and Wang; Genc et al.; SamavatiJame et al.; Ma et al. [14–17, 20–37]	Follow-up ranged from 12 months to 12 years. Most studies reported short- or medium-term outcomes, mainly between 12 and 36 months. Longer-term outcomes were available only in a limited number of studies, including SamavatiJame et al., Anitua et al., Ainiwaer et al., and Ronchetti et al.	Long-term evidence was limited and mainly derived from case reports, case series, or retrospective analyses.
Tooth survival, success, and function	Reported across most studies, including Hamamoto et al.; Marques-Ferreira et al.; Chaudhary et al.; Miura et al.; Keranmu et al.; Gonzalez-Ocasio and Stevens; Alkofahi et al.; Gaviño-Orduña et al.; Mena-Álvarez et al.; Rey Lescure et al.; Anitua et al.; Sakhariya et al.; Alijani et al.; Pedrinaci et al.; Shetty et al.; Ainiwaer et al.; Ainiwaer and Wang; Genc et al.; SamavatiJame et al.; and Ma et al. [14–17, 20–28, 30–36]	Most studies reported functional transplanted teeth at follow-up, with favorable survival or success when these outcomes were assessed. Comparative studies generally reported high survival or success in both test and control groups.	Failures were reported in some studies, mainly related to persistent apical periodontitis, root resorption, bone loss, or transplant extraction, particularly in Marques-Ferreira et al., Adamska et al., Ainiwaer et al., Genc et al., and Ronchetti et al. [15, 17, 29, 36, 37]
Pulp vitality, sensibility, and root development	Mainly reported in studies involving immature donor teeth with open apices: Gonzalez-Ocasio and Stevens; Alkofahi et al.; Rey Lescure et al.; Anitua et al.; Adamska et al.; SamavatiJame et al.; and Ma et al. [23, 26, 29, 31, 32, 34, 35]	Immature donor teeth were commonly monitored without immediate endodontic treatment. Favorable findings included maintenance or recovery of pulp sensibility, continued root development, and apical closure.	Pulp necrosis and subsequent failure were reported in Adamska et al. Outcome assessment methods were not standardized across studies.
Periodontal status and mobility	Reported in studies assessing probing depth, attachment level, mobility, periodontal stability, or function, including Hamamoto et al.; Marques-Ferreira et al.; Keranmu et al.; Gonzalez-Ocasio and Stevens; Sakhariya et al.; Pedrinaci et al.; Shetty et al.; Ainiwaer et al.; Ainiwaer and Wang; Genc et al.; and Ma et al. [15–17, 20, 27, 30, 32–34, 36]	Most studies described acceptable periodontal parameters, physiologic mobility, and clinical function when these outcomes were assessed.	Increased mobility, periodontal bone loss, or unfavorable periodontal healing were reported inconsistently and mainly in studies with complications or failures, including Sakhariya et al., Adamska et al., Ainiwaer et al., and Genc et al. [17, 27, 29, 36]
Periapical healing, PDL space, and bone healing	Reported in studies assessing periapical healing, radiographic bone fill, PDL space, lamina dura, or bone regeneration, including Miura et al.; Chaudhary et al.; Marques-Ferreira et al.; Keranmu et al.; Alkofahi et al.; Gaviño-Orduña et al.; Mena-Álvarez et al.; Rey Lescure et al.; Anitua et al.; Adamska et al.; Pedrinaci et al.; Shetty et al.; Ainiwaer and Wang; and Ma et al. [15, 16, 20–26, 29, 30, 32, 33, 35]	Favorable radiographic findings included periapical healing, maintenance of PDL space, lamina dura formation, bone fill, and absence of recurrent pathology.	Persistent periapical pathology or radiolucency was reported in some failed or complicated cases, including Marques-Ferreira et al. and Adamska et al. [15, 29]
Root resorption and ankylosis	Reported across clinical and radiographic follow-up assessments, including Marques-Ferreira et al.; Chaudhary et al.; Miura et al.; Keranmu et al.; Alkofahi et al.; Gaviño-Orduña et al.; Mena-Álvarez et al.; Rey Lescure et al.; Anitua et al.; Sakhariya et al.; Alijani et al.; Adamska et al.; Pedrinaci et al.; Shetty et al.; Ainiwaer et al.; Ainiwaer and Wang; Genc et al.; SamavatiJame et al.; and Ma et al. [15–17, 20–33, 35, 36]	Most studies reported absence of progressive inflammatory resorption, replacement resorption, or ankylosis in surviving teeth.	Inflammatory root resorption, replacement root resorption, and ankylosis were among the most relevant complications, particularly in Marques-Ferreira et al., Adamska et al., Ainiwaer et al., Genc et al., and Ronchetti et al. [15, 17, 29, 36, 37]
Patient-centered outcomes	Reported in a limited number of studies, mainly Hamamoto et al.; Ma et al.; Anitua et al.; Pedrinaci et al.; and Ainiwaer et al. [14, 20, 32, 35, 36]	Patient-centered outcomes were rarely assessed. When reported, they included patient satisfaction, esthetics, function, or treatment acceptance.	These outcomes were not standardized and were absent from most included studies.

*ATT* autotransplantation, *CGF* concentrated growth factor, *DFDBA* demineralized freeze-dried bone allograft, *EMD* enamel matrix derivative, *EPT* electric pulp test, *PDL* periodontal ligament, *PRF* platelet-rich fibrin, *PRGF* plasma rich in growth factors, *PRP* platelet-rich plasma, *RCT* root canal treatment

The most frequently reported outcomes were tooth survival or success, pulp vitality or sensibility, continued root development or apical closure in immature teeth, periodontal probing depth, mobility, periapical healing, PDL space, root resorption, ankylosis, and radiographic bone healing. These outcomes were reported across studies [14–17, 20–37]. Patient-centered outcomes were rarely reported and were limited mainly to patient satisfaction, esthetics, function, or treatment acceptance in a small number of studies [14, 20, 32, 35, 36].

Overall, most sources reported uneventful healing and functional transplanted teeth at follow-up, with physiologic mobility and acceptable periodontal parameters when these outcomes were assessed. In studies involving immature teeth, favorable findings commonly included maintenance or recovery of pulp sensibility, continued root development, and apical closure [23, 31, 32, 34, 35]. In studies involving mature teeth, favorable outcomes were more often reported in relation to periodontal stability, absence of progressive resorption, periapical healing, and function after endodontic treatment [14, 16, 20, 22, 24, 25, 28, 30, 33, 36].

Comparative clinical studies [15–17] reported high success or survival rates in both test and control groups, although the measured benefits of adjuncts varied by study and outcome. The most relevant complications were pulp necrosis, inflammatory root resorption, replacement root resorption, ankylosis, persistent periapical pathology, increased mobility, bone loss, and transplant failure [15, 17, 29, 36, 37]. These complications were reported inconsistently, and outcome definitions were not standardized across studies.

### Critical appraisal

**Fig. 2:**
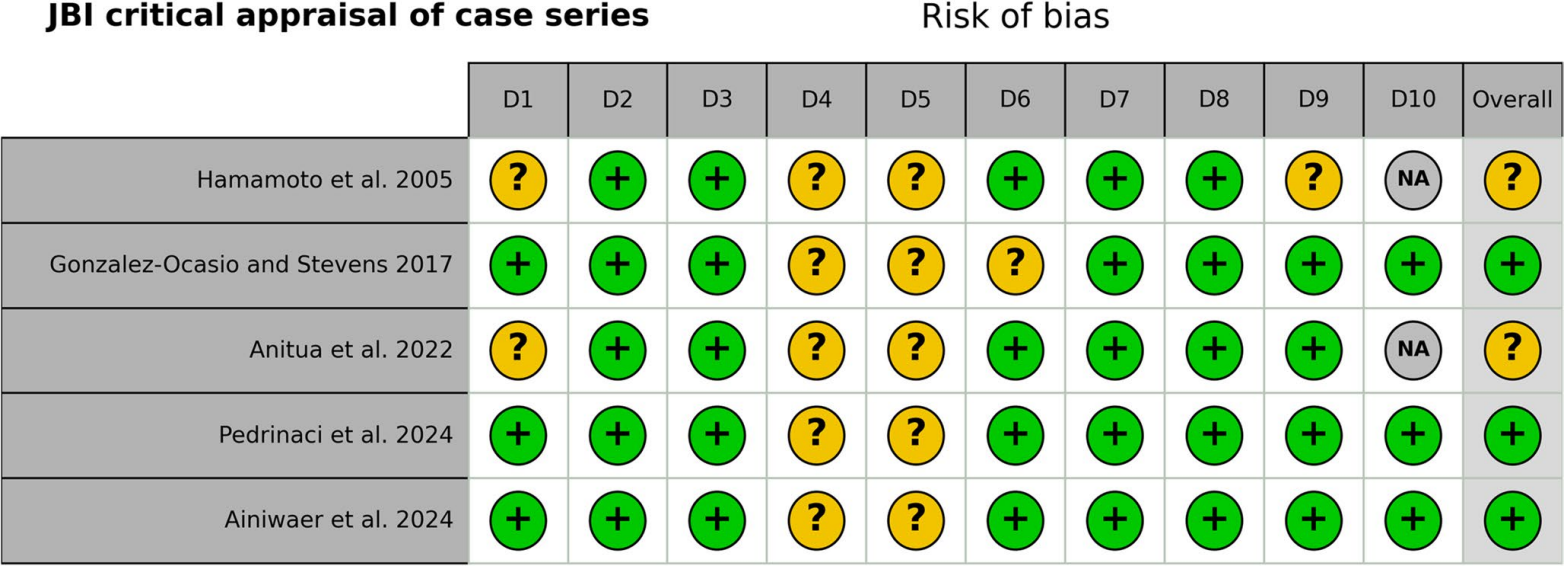
JBI critical appraisal of case series. The figure summarizes domain-level judgments for the included case series. Q1, clear inclusion criteria; Q2, standard and reliable measurement of the condition; Q3, valid methods for identifying the condition; Q4, consecutive inclusion of participants; Q5, complete inclusion of participants; Q6, reporting of participant demographics; Q7, reporting of clinical information; Q8, reporting of outcomes or follow-up; Q9, reporting of presenting site or clinic information; Q10, appropriate statistical analysis. Green indicates yes; yellow indicates unclear; gray indicates not applicable. Abbreviations: JBI, Joanna Briggs Institute; NA, not applicable

**Fig. 3:**
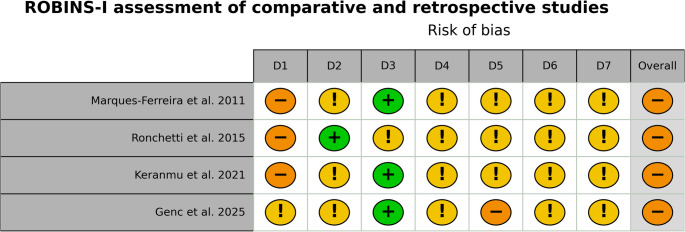
ROBINS-I risk-of-bias assessment of comparative and retrospective studies. D1, confounding; D2, selection of participants; D3, classification of interventions; D4, deviations from intended interventions; D5, missing data; D6, measurement of outcomes; D7, selection of the reported result. Green indicates low risk of bias; yellow indicates moderate risk of bias; orange indicates serious risk of bias; red indicates critical risk of bias; gray indicates no information. Abbreviations: NI, no information; ROBINS-I, Risk Of Bias In Non-randomized Studies of Interventions

Critical appraisal was performed to characterize the methodological rigor of the included evidence and to support the interpretation of the mapped findings. No study was excluded based on appraisal results, consistent with the descriptive purpose of this scoping review. The critical appraisal results are summarized in Table 5, and the detailed JBI and ROBINS-I assessments are provided in Online Resource 1, Supplementary Tables S6–S8. The JBI appraisal of case series is presented in Fig. 2, the ROBINS-I domain-level risk-of-bias assessment for comparative and retrospective studies is presented in Fig. 3, and the JBI appraisal of case reports is provided in Online Resource 1, Supplementary Figure S1.

**Table 5:** Summary of critical appraisal and implications for interpretation

Evidence group	Included sources	Appraisal tool	Main appraisal findings	Implication for interpretation
Case reports	Chaudhary et al.; Miura et al.; Alkofahi et al.; Gaviño-Orduña et al.; Mena-Álvarez et al.; Rey Lescure et al.; Sakhariya et al.; Alijani et al.; Adamska et al.; Shetty et al.; Ainiwaer and Wang; SamavatiJame et al.; Ma et al. [21–33]	JBI Critical Appraisal Checklist for Case Reports	Most case reports provided adequate information on patient characteristics, clinical presentation, diagnostic assessment, intervention, follow-up, adverse events, and clinical take-home message.	Useful for mapping adjunct protocols, clinical feasibility, and reported outcomes, but not suitable for determining adjunct efficacy.
Case series	Hamamoto et al.; Gonzalez-Ocasio and Stevens; Anitua et al.; Pedrinaci et al.; Ainiwaer et al. [14, 20, 34–36]	JBI Critical Appraisal Checklist for Case Series	Case series generally described clinical procedures and outcomes, but some studies had unclear consecutive or complete inclusion of participants and heterogeneous follow-up schedules.	Useful for identifying clinical patterns and procedural variability, but limited for causal inference.
Comparative and retrospective studies	Marques-Ferreira et al.; Keranmu et al.; Genc et al.; Ronchetti et al. [15–17, 37]	ROBINS-I	All comparative or retrospective studies were judged as having an overall serious risk of bias. The most problematic domains were confounding and, in some studies, missing data and outcome measurement.	Findings comparing adjunctive and non-adjunctive protocols should be interpreted cautiously and considered hypothesis-generating.

*JBI* Joanna Briggs Institute, *ROBINS-I* Risk Of Bias In Non-randomized Studies of Interventions

The case reports [21–33] generally provided adequate descriptions of clinical presentation, diagnostic assessment, intervention protocols, follow-up, adverse events, and clinical take-home messages. However, their single-patient or limited-case design and absence of comparison groups limit causal interpretation. The case series [14, 20, 34–36] also generally described clinical procedures and outcomes adequately. However, several had unclear reporting regarding consecutive or complete inclusion of participants, small samples, heterogeneous follow-up schedules, and non-standardized outcome definitions. The comparative clinical studies [15–17], as well as the retrospective analysis by Ronchetti et al. [37], provided more clinically relevant comparisons, but all were judged as having an overall serious risk of bias.

Confounding was the main methodological concern in the comparative and retrospective studies, largely because adjunct use was intertwined with differences in root maturity, donor tooth type, recipient-site condition, surgical complexity, endodontic strategy, stabilization protocol, operator-related factors, and additional co-interventions. Missing data was a moderate concern in Marques-Ferreira et al. [15], Ronchetti et al. [37], and Keranmu et al. [16], and a serious concern in Genc et al. [17]. Outcome measurement was a moderate concern across all comparative and retrospective studies [15–17, 37]. Consequently, favorable clinical and radiographic outcomes should not be interpreted as evidence of an independent adjunct effect. Comparative findings remain hypothesis-generating and should be interpreted cautiously.

## Discussion

The main finding of this scoping review was that biological adjuncts used in ATT are feasible but highly heterogeneous. The available evidence mainly involved platelet-derived preparations and EMD, applied across different clinical scenarios and procedural protocols [20, 38, 39]. Most included sources reported favorable clinical and radiographic outcomes; however, the evidence base was predominantly composed of case reports and case series, with few comparative studies. Therefore, the current literature should be interpreted as evidence of clinical feasibility and biological plausibility rather than evidence of efficacy or superiority of any specific adjunctive protocol. The independent effect of biological adjuncts remains uncertain and should be interpreted in relation to root maturity, surgical handling, recipient-site condition, stabilization, and endodontic management [16, 20, 21, 28, 34].

### Biological adjuncts

#### Platelet-derived preparations

The growing interest in autologous PCs is related to their biological plausibility and applicability as adjunctive biomaterials in tissue healing and regeneration. Historically, these products include PRP [40], PRGF [41], L-PRF [42], and, more recently, CGF [43]. These preparations differ in centrifugation protocol, fibrin architecture, leukocyte content, platelet concentration, handling properties, and growth-factor release profile. Their proposed biological role is mainly related to the provision of a fibrin scaffold and a reservoir of bioactive mediators that may support cell migration, angiogenesis, PDL repair, osteogenesis, and cellular proliferation and differentiation [39].

In the included studies, platelet-derived preparations were more frequently used in complex recipient sites, particularly in areas with pre-existing bone defects, infection-related defects, or extraction sockets associated with loss of supporting bone [16, 21, 22, 29, 30, 35, 36]. In these contexts, they may provide a biologically favorable environment for recipient-site healing and adaptation of the transplanted tooth. However, because these adjuncts were often used together with other surgical and regenerative strategies, including grafting materials, membranes, digital planning, or specific endodontic protocols, their independent effect remains difficult to determine. The available evidence also does not allow a direct comparison between different platelet-derived preparations or PRF generations. Although PRF, PRP, PRGF, and CGF have distinct biological and handling characteristics, the included studies differed substantially in root maturity, recipient-site condition, application method, stabilization, endodontic management, and follow-up. Therefore, no conclusion can be drawn regarding the superiority of one platelet-derived adjunct over another in tooth autotransplantation.

In teeth with incomplete root formation, this effect may be particularly relevant, since aggregates such as PRF may favor a microenvironment more conducive to tissue repair [44, 45]. Studies with immature teeth reported maintenance of pulp vitality, as well as root development and apical closure, even though the underlying biological mechanisms were not directly evaluated [23, 26, 31, 32, 34, 35]. These outcomes should also be interpreted considering the intrinsic biological potential of immature teeth for pulp revascularization and continued root formation. Regarding the mode of application, platelet-derived preparations were most frequently placed within the recipient socket, suggesting a potential role in supporting socket healing and biological adaptation of the transplanted tooth. Nevertheless, this application strategy should not be interpreted as predictably associated with clinical success.

In Adamska et al. [29], despite the use of A-PRF associated with an autogenous dentin graft, the case evolved with pulp necrosis, external cervical inflammatory resorption, and transplant failure, culminating in the extraction of the tooth after 12 months. This finding suggests that platelet aggregates should not be interpreted as isolated determinants of clinical success, despite their biological plausibility and use as adjunctive strategies. In teeth with complete root formation, their potential benefit becomes even more difficult to isolate, since endodontic treatment was part of the therapeutic protocol in several studies, whether early, planned, or performed according to clinical progression [16, 25, 30, 36]. Therefore, their role appears to be more associated with biological support for repair of the recipient site, favoring alveolar healing and integration of the transplanted tooth, than with pulp outcomes per se.

Recently, horizontal centrifugation has been proposed to optimize the distribution and concentration of cells throughout the PRF clot while enhancing the release of growth factors [46, 47]. Indeed, preclinical evidence suggests that this technique is superior to fixed-angle centrifugation protocols in promoting bone and cementum formation [48, 49]. However, to date, no study has evaluated the use of PCs produced by horizontal centrifugation as an adjunct to ATT.

#### EMD

The use of EMD in tooth autotransplantation is based on its biological plausibility as a modulator of periodontal wound healing. When applied to the donor root surface, EMD may mimic biological events involved in root development, particularly cementum formation, PDL repair, and periodontal regeneration [38, 50, 51]. This rationale is clinically relevant in ATT, since preservation and repair of the PDL are central determinants of periodontal healing, root-surface integrity, and prevention of complications such as replacement resorption and ankylosis.

In the included studies, EMD was generally applied topically to the donor root surface before transplantation and was mainly reported in mature or mixed-root populations [14, 15, 20, 37]. Pedrinaci et al. [20] provide one of the most direct clinical descriptions of EMD use in ATT, reporting high survival and success rates after a standardized digital protocol with adjunctive EMD application in molars with complete root formation. However, the isolated effect of EMD remains difficult to determine, since this study had no control group and combined several potentially favorable factors, including digital planning, standardized socket preparation, and prophylactic endodontic treatment. In one 30-year-old patient, clinical stability was maintained for two years after EMD-assisted ATT, with favorable periodontal and prosthetic conditions; however, this finding should be interpreted cautiously because it derives from a single case report [14].

Although some studies compared EMD with saline solution, their interpretation is limited by heterogeneous surgical techniques, different stages of root development, and diverse clinical indications, making it difficult to isolate the specific effect of EMD [15]. In addition, in retrospective analyses, no significant association was identified between its use and long-term success, a finding that should be interpreted with caution given the observational design, the influence of the surgeon on outcomes, and the small number of events [37]. Therefore, EMD appears to be a biologically plausible adjunct for periodontal healing in ATT, but current evidence remains insufficient to demonstrate an independent clinical benefit or superiority over standard protocols.

### Root maturity and endodontic implications

Root maturity is a key factor in ATT outcomes. Teeth with open apices have greater potential for pulp revascularization and continued root development, so transplantation is generally more favorable when root formation is incomplete (about one-half to two-thirds) [52]. In these cases, immediate endodontic treatment is usually not required, and follow-up focuses on pulp sensibility, root development, apical closure, and absence of inflammation. In contrast, teeth with closed apices have limited regenerative potential and often require early endodontic treatment [52]. However, ATT can be successful in both immature and mature teeth, although immature teeth tend to show fewer complications [52, 53].

In the included studies, teeth with open apices were typically managed without immediate RCT, with monitoring focused on pulpal and radiographic outcomes. This approach was reported in studies using platelet-derived preparations [23, 26, 29, 31, 32, 34, 35]. However, these results should be interpreted cautiously, as immature teeth already have a natural capacity for revascularization and root maturation [52, 53]. Marques-Ferreira et al. [15] provided the clearest comparison, showing that RCT was more frequent in closed-apex teeth, while pulp revascularization occurred mainly in open-apex teeth. Similarly, studies on immature third molars often reported no need for endodontic treatment and favorable outcomes [23, 26, 31, 32, 34, 35]. However, no study demonstrated that biological adjuncts independently improved root development or revascularization.

In teeth with complete root formation, interpretation is more limited. Mature teeth were often treated with planned or early root canal therapy, particularly in studies using EMD, CGF, PRF, or PRGF [14, 16, 20, 24, 25, 27, 28, 30, 33, 36]. Although favorable outcomes were reported, they were influenced by multiple factors, making it difficult to isolate the effect of biological adjuncts. Comparative studies also support a cautious interpretation. Marques-Ferreira et al. [15] (EMD vs. saline) and Genc et al. [17] (PRP) did not show clear superiority of adjuncts. Overall, root maturity appears to be a more consistent prognostic factor than the use of biological adjuncts, which may support healing but do not clearly alter outcomes based on root development stage.

### Surgical variables

It is worth noting that operator experience appears to influence treatment success. In one of the included studies, the most experienced surgeon presented only one failure after more than 8 years of follow-up, whereas the less experienced surgeon recorded four failures [37]. These findings reinforce the possible importance of the learning curve for ATT success. There is a tendency for greater root maturity to be associated with less favorable pulp and periodontal outcomes, possibly due to the lower potential for revascularization and the greater probability of needing endodontic treatment [9, 52].

In general, the included studies suggest that ATT success is repeatedly associated with surgical fundamentals, rather than with the choice of the biological adjunct in isolation. Among these factors, atraumatic extraction of the donor tooth, reduction of extraoral time, minimal manipulation of the root surface, careful preparation of the recipient site, achievement of primary stability, and adequate infection control and debridement when indicated are particularly relevant [2, 13]. Since these variables frequently coexist with the use of adjuncts, it becomes difficult to isolate their specific effect and directly compare results between studies. The condition of the recipient site was another important source of heterogeneity. Biological adjuncts were frequently used in defective, infected, or reconstructed sites, as reported by Chaudhary et al. [21] in a large residual bony defect, Miura et al. [22] in an alveolar cleft, Keranmu et al. [16] in inflamed recipient sites, Adamska et al. [29] in a defective socket, and Ainiwaer et al. [36] in sites with compromised supporting bone. In these scenarios, adjuncts may have a stronger biological rationale as part of a broader regenerative strategy. However, because they were commonly combined with grafts, membranes, digital planning, or additional surgical procedures, their specific contribution remains uncertain.

Stabilization protocols were heterogeneous and often incompletely reported. Short-term suture-supported, non-rigid, flexible, or semi-rigid stabilization was described [14–17, 20, 25, 26, 30, 33, 34]. Fiberglass band stabilization was reported [16, 36]. However, key details such as wire diameter, passive adaptation, splint rigidity, duration, and criteria for removal were frequently missing. Only Sakhariya et al. [27] and Adamska et al. [29] clearly reported wire diameter, using 24G stainless-steel wire and 0.016-inch stainless-steel wire, respectively. This incomplete reporting limits the interpretation of stabilization as a prognostic variable.

### Limitations of the evidence base

The available literature presents important limitations. The main one is the predominance of non-comparative studies, especially case reports and case series, which reduces the robustness of clinical inferences. In addition, many studies included small samples, heterogeneous protocols, and variable follow-up times. Although the outcomes evaluated were relatively similar between studies, including pulp vitality, mobility, probing depth, resorption, ankylosis, and radiographic findings, measurement methods, success criteria, and follow-up intervals were not uniform. This heterogeneity limits comparability between studies and makes critical synthesis of the available evidence difficult [13]. This interpretation is reinforced by the critical appraisal. Comparative and retrospective studies were judged as having serious risk of bias, mainly due to confounding, missing data, and outcome measurement limitations. In these studies, adjunct use was frequently intertwined with root maturity, recipient-site condition, endodontic strategy, stabilization protocol, operator experience, and co-interventions. Therefore, favorable outcomes should not be interpreted as evidence of an independent adjunct effect.

Another relevant limitation is the incomplete description of potentially critical procedural variables, such as extraoral time, splint rigidity, wire diameter, analgesic and antibiotic protocols, precise indication for endodontic treatment, and the method used for assessment of pulp vitality. In some studies, the simultaneous presence of multiple interventions, such as digital planning, bone grafts, membranes, and combined biomaterials, further complicates the interpretation of the isolated effect of the biological adjunct. In addition, if the review protocol was not prospectively registered, this should also be acknowledged as a methodological limitation.

### Implications for clinical practice and research

From a clinical point of view, biological adjuncts have been reported mainly in more complex scenarios, such as bone defects, compromised sockets, or chronic periapical lesions. However, the current evidence still does not support the consistent superiority of one adjunctive protocol over another. Thus, root maturity should continue to be one of the main factors guiding the endodontic strategy, while short-term semi-rigid splinting remains the approach most frequently adopted in clinical studies [11].

Patient-centered outcomes also deserve greater attention. Although available data suggest favorable esthetic satisfaction and general acceptance of ATT in most patients [54], aspects such as function, esthetics, discomfort, treatment acceptability, overall satisfaction, quality of life, and cost-effectiveness have been assessed in a limited and poorly standardized manner [36]. This is particularly relevant because biological adjuncts may increase chair time, procedural complexity, material costs, or require venous blood collection.

The feasibility of platelet-derived preparations should also be considered, especially in children and adolescents. Since PRF, PRP, PRGF, and CGF require venous blood collection, their use depends on patient cooperation, caregiver consent, venipuncture feasibility, and trained personnel. When blood collection is not feasible, the potential benefit of these adjuncts should be weighed against the additional procedural burden. Future studies should therefore evaluate whether adjuncts provide measurable benefits from the patient’s perspective, beyond clinical and radiographic outcomes.

For future research, the development of a minimum set of variables to be reported in a standardized manner appears essential, including stage of root formation, extraoral time, type and duration of splinting, endodontic protocol, method of vitality assessment, and definition of success [13]. Comparative studies are also necessary, ideally in well-defined clinical scenarios, such as healthy versus compromised recipient sites, immature versus mature donor teeth, and adjunctive versus non-adjunctive protocols, with longer follow-up and standardized clinical, radiographic, and patient-reported outcomes.

## Conclusion

Biological adjuncts in tooth autotransplantation have been reported as clinically feasible in selected studies. Nevertheless, the available evidence remains weak, as it is largely derived from case reports, case series, and studies lacking comparator groups. This limits the interpretation of their true clinical effect and prevents definitive conclusions regarding their independent benefit. Therefore, the use of these adjuncts should be considered with caution until well-designed comparative studies clarify their role in improving the predictability of tooth autotransplantation.

## Supplementary Information

Below is the link to the electronic supplementary material.

Supplementary Material 1 (DOCX 1.96 MB)

## Data Availability

All data generated or analyzed during this study are included in this published article and its supplementary information files.
