# Supplementary material for: The use of biologic adjuncts in tooth autotransplantation: a scoping review
Source: Oral Maxillofac Surg. 2026 Aug 7;30(1):127. doi: 10.1007/s10006-026-01613-w (PMC13451306; doi:10.1007/s10006-026-01613-w)
Supplement: Supplementary file 1 [file 10006_2026_1613_MOESM1_ESM.docx]

**Supplementary material**

**Title:** The Use of Biologic Adjuncts in Tooth Autotransplantation: A Scoping Review

**Authors**

Edgar Daniel Vargas-Quirogaᵃ, Brenda Carolina Pattigno Foreroᵇ, Ana Carolina Duarte Firminoᵃ, Debora S. F. Sávioᵃ, Carolina Alves Freiria Oliveiraᶜ, Isabel Schausltz Pereira Faustinoᵈ, Arthur Belem Novaes Jrᵃ

**Affiliations**

ᵃ Department of Oral and Maxillofacial Surgery and Periodontology, Ribeirão Preto School of Dentistry, University of São Paulo, Ribeirão Preto, São Paulo, Brazil. ᵇ Department of Pediatric Dentistry, Ribeirão Preto School of Dentistry, University of São Paulo, Ribeirão Preto, São Paulo, Brazil. ᶜ Department of Dental Materials and Prosthodontics, Ribeirão Preto School of Dentistry, University of São Paulo, Ribeirão Preto, São Paulo, Brazil. ᵈ Department of Stomatology, Public Oral Health and Forensic Dentistry, Ribeirão Preto School of Dentistry, University of São Paulo, Ribeirão Preto, São Paulo, Brazil.

**Contents**

**Page 1. PCC framework**

**Page 2. Conceptual search blocks and database-specific strategies**

**Page 6 . Supplementary tables and figures**

**Supplementary Table S1.** Reports excluded after full-text assessment and reasons for exclusion

**Supplementary Table S2.** Detailed study characteristics and clinical categorization of included sources

**Supplementary Table S3.** Detailed biological adjuncts and procedural protocols of included sources

**Supplementary Table S4.** Adjunct preparation protocols, centrifugation parameters, and application sites.

**Supplementary Table S5.** Detailed follow-up and reported outcomes of included sources

**Supplementary Table S6.** JBI Critical Appraisal Checklist for Case Reports.

**Supplementary Table S7.** JBI Critical Appraisal Checklist for Case Series.

**Supplementary Table S8.** ROBINS-I assessment for comparative and retrospective studies.

**Supplementary Figure S1.** JBI critical appraisal of case reports..

1. **PCC framework**

**Objective**

To map biological adjuncts used in human tooth autotransplantation, describe their application protocols, and summarize reported clinical and radiographic outcomes.

PCC framework

- Population (P): Individuals undergoing tooth autotransplantation.
- Concept (C): Use of biological adjuncts during the autotransplant procedure, including but not limited to enamel matrix derivative (EMD), platelet concentrates (PRF and subtypes, PRP, PRGF, CGF, platelet lysate), platelet-derived growth factor (PDGF or rhPDGF), fibroblast growth factor 2 (FGF-2 or trafermin), bone morphogenetic proteins (BMP-2, BMP-7), transforming growth factor beta (TGF-β), vascular endothelial growth factor (VEGF), insulin-like growth factor (IGF), and hyaluronic acid.
- Context (C): Clinical dental practice, in any setting or level of care.

**Research question**

Which biological adjuncts have been used in human tooth autotransplantation, how are they applied, and which clinical and radiographic outcomes are reported?

**Information sources and search limits**

Electronic searches were conducted in the following databases:

1. MEDLINE (via PubMed)
2. Embase
3. Scopus
4. Web of Science
5. LILACS via the Virtual Health Library

All databases were searched from inception to 21 June 2026, with no initial restriction on year of publication. No language filters were applied at the search stage. Search strategies were tailored to the syntax and indexing of each database, combining controlled vocabulary (MeSH, Emtree, DeCS) and free-text terms.

ClinicalTrials.gov was searched on 28 June 2026 as a complementary registry source. The broad ATT-related search retrieved 12 records, and the adjunct-specific search retrieved 6 records. Registry records were screened using the same eligibility criteria as the main review. Records without published clinical outcome data were not included as sources of evidence; when a registry record corresponded to an already identified published article, the article was used as the source of evidence.

3. **Conceptual search blocks**

Search terms were structured in two main conceptual blocks::

| Population | Concept |
| --- | --- |
| ( ("Tooth Transplantation"[Mesh] OR "Tooth Replantation"[Mesh]) OR ( (autotransplant*[tiab] OR "tooth autotransplant*"[tiab] OR "dental autotransplant*"[tiab]) AND (tooth*[tiab] OR dental*[tiab]) ) OR ( (autologous[tiab] OR autogen*[tiab]) AND (tooth*[tiab] OR dental*[tiab]) AND (transplant*[tiab] OR transplantat*[tiab]) ) ) | ( ("Enamel Matrix Derivative"[Mesh] OR "enamel matrix derivative*"[tiab] OR "enamel matrix protein*"[tiab] OR emdogain[tiab] OR EMD[tiab]) OR ("Platelet-Rich Plasma"[Mesh] OR "platelet rich plasma"[tiab] OR "platelet-rich plasma"[tiab] OR PRP[tiab]) OR ("platelet rich fibrin"[tiab] OR "platelet-rich fibrin"[tiab] OR PRF[tiab] OR "leukocyte and platelet rich fibrin"[tiab] OR L-PRF[tiab] OR A-PRF[tiab] OR i-PRF[tiab]) OR ("plasma rich in growth factor*"[tiab] OR PRGF[tiab]) OR ("concentrated growth factor*"[tiab] OR CGF[tiab]) OR ("platelet lysate"[tiab] OR "platelet concentrate*"[tiab]) OR ("Platelet-Derived Growth Factor"[Mesh] OR "platelet derived growth factor"[tiab] OR PDGF[tiab] OR "PDGF-BB"[tiab] OR rhPDGF[tiab] OR becaplermin[tiab] OR "GEM 21S"[tiab]) OR ("Fibroblast Growth Factors"[Mesh] OR "fibroblast growth factor*"[tiab] OR FGF[tiab] OR "FGF-2"[tiab] OR bFGF[tiab] OR trafermin[tiab]) OR ("Bone Morphogenetic Proteins"[Mesh] OR "bone morphogenetic protein*"[tiab] OR BMP[tiab] OR "BMP-2"[tiab] OR "BMP-7"[tiab]) OR ("Transforming Growth Factor beta"[Mesh] OR "transforming growth factor*"[tiab] OR "TGF beta"[tiab] OR TGF-beta[tiab]) OR ("vascular endothelial growth factor"[tiab] OR VEGF[tiab]) OR ("insulin like growth factor"[tiab] OR "insulin-like growth factor"[tiab] OR IGF[tiab]) OR ("Hyaluronic Acid"[Mesh] OR "hyaluronic acid"[tiab] OR hyaluronan[tiab] OR hyaluronate[tiab]) ) |

The two blocks were combined using the Boolean operator AND. Synonyms and related terms within each block were combined using OR. Database-specific controlled vocabulary and search fields were adapted according to each database.

**Database specific strategies**

1 Pubmed

| Pubmed | Data |
| --- | --- |
| #1  ( ("Tooth"[MeSH Terms] AND "Transplantation, Autologous"[MeSH Terms]) OR autotransplant*[All Fields] OR "auto-transplant*"[All Fields] OR ("tooth transplant*"[All Fields] OR "teeth transplant*"[All Fields]) OR ("tooth autotransplant*"[All Fields] OR "teeth autotransplant*"[All Fields]) OR ("autogenous tooth transplant*"[All Fields] OR "autogenous teeth transplant*"[All Fields]) OR ("autologous tooth transplant*"[All Fields] OR "autologous teeth transplant*"[All Fields]) )  #2  ( "Platelet-Rich Fibrin"[MeSH Terms] OR "Platelet-Rich Plasma"[MeSH Terms] OR "Platelet-Derived Growth Factor"[MeSH Terms] OR "Becaplermin"[MeSH Terms] OR "Fibroblast Growth Factors"[MeSH Terms] OR "Fibroblast Growth Factor 2"[MeSH Terms] OR "Fibroblast Growth Factor 7"[MeSH Terms] OR "Bone Morphogenetic Proteins"[MeSH Terms] OR "Bone Morphogenetic Protein 2"[MeSH Terms] OR "Transforming Growth Factor beta"[MeSH Terms] OR "Vascular Endothelial Growth Factor A"[MeSH Terms] OR "Insulin-Like Growth Factor I"[MeSH Terms] OR "Hyaluronic Acid"[MeSH Terms] OR emdogain[All Fields] OR "enamel matrix derivative"[All Fields] OR "enamel matrix derivatives"[All Fields] OR "enamel matrix proteins"[All Fields] OR EMD[All Fields] OR PRF[All Fields] OR "leukocyte- and platelet-rich fibrin"[All Fields] OR L-PRF[All Fields] OR A-PRF[All Fields] OR i-PRF[All Fields] OR PRP[All Fields] OR "plasma rich in growth factors"[All Fields] OR PRGF[All Fields] OR CGF[All Fields] OR "concentrated growth factor"[All Fields] OR "concentrated growth factors"[All Fields] OR "platelet lysate"[All Fields] OR PDGF-BB[All Fields] OR "platelet-derived growth factor BB"[All Fields] OR rhPDGF[All Fields] OR "GEM 21S"[All Fields] OR trafermin[All Fields] OR "recombinant fibroblast growth factor 2"[All Fields] OR rhFGF-2[All Fields]  #1 AND #2  Link:  <https://pubmed.ncbi.nlm.nih.gov/?term=longquerye56c5d8c5f58e4501eb1&sort=relevance> | 21/06/2026 |
| Results | 166 |

2 Embase:

| Embase | Data |
| --- | --- |
| #1 'tooth transplantation'/exp OR 'tooth transplantation' OR autotransplant*:ti,ab,kw OR 'auto-transplant*':ti,ab,kw OR 'tooth transplant*':ti,ab,kw OR 'teeth transplant*':ti,ab,kw OR 'tooth autotransplant*':ti,ab,kw OR 'teeth autotransplant*':ti,ab,kw OR 'autogenous tooth transplant*':ti,ab,kw OR 'autogenous teeth transplant*':ti,ab,kw OR 'autologous tooth transplant*':ti,ab,kw OR 'autologous teeth transplant*':ti,ab,kw  #2 'platelet rich fibrin'/exp OR 'platelet rich plasma'/exp OR 'platelet derived growth factor'/exp OR 'becaplermin'/exp OR 'fibroblast growth factor'/exp OR 'fibroblast growth factor 2'/exp OR 'fibroblast growth factor 7'/exp OR 'bone morphogenetic protein'/exp OR 'bone morphogenetic protein 2'/exp OR 'transforming growth factor beta'/exp OR 'vascular endothelial growth factor a'/exp OR 'insulin like growth factor 1'/exp OR 'hyaluronic acid'/exp OR emdogain:ti,ab,kw OR 'enamel matrix derivative*':ti,ab,kw OR 'enamel matrix protein*':ti,ab,kw OR emd:ti,ab,kw OR prf:ti,ab,kw OR 'leukocyte- and platelet-rich fibrin':ti,ab,kw OR 'l prf':ti,ab,kw OR 'a prf':ti,ab,kw OR 'i prf':ti,ab,kw OR prp:ti,ab,kw OR 'plasma rich in growth factors':ti,ab,kw OR prgf:ti,ab,kw OR cgf:ti,ab,kw OR 'concentrated growth factor*':ti,ab,kw OR 'platelet lysate':ti,ab,kw OR 'pdgf bb':ti,ab,kw OR 'platelet-derived growth factor bb':ti,ab,kw OR rhpdgf:ti,ab,kw OR 'gem 21s':ti,ab,kw OR trafermin:ti,ab,kw OR 'recombinant fibroblast growth factor 2':ti,ab,kw OR 'rhfgf 2':ti,ab,kw  #1 AND #2 | 21/06/2026 |
| Results | 216 |

3 Web of Science

| Web of Science | Data |
| --- | --- |
| All fields:  autotransplant* OR "auto-transplant*" OR "tooth transplant*" OR "teeth transplant*" OR "tooth autotransplant*" OR "teeth autotransplant*" OR "autogenous tooth transplant*" OR "autogenous teeth transplant*" OR "autologous tooth transplant*" OR "autologous teeth transplant*"  **AND**  All fields:  "platelet-rich fibrin" OR PRF OR "leukocyte- and platelet-rich fibrin" OR L-PRF OR A-PRF OR i-PRF OR "platelet-rich plasma" OR PRP OR "plasma rich in growth factors" OR PRGF OR "concentrated growth factor*" OR CGF OR "platelet lysate" OR "platelet-derived growth factor" OR PDGF OR "PDGF-BB" OR rhkgf OR "GEM 21S" OR becaplermin OR "fibroblast growth factor*" OR FGF OR "FGF-2" OR "basic fibroblast growth factor" OR bFGF OR terraferma OR "bone morphogenetic protein*" OR BMP OR "BMP-2" OR "BMP-7" OR "transforming growth factor*" OR "TGF beta" OR TGF-beta OR "vascular endothelial growth factor" OR VEGF OR "insulin-like growth factor" OR IGF OR "hyaluronic acid" OR hyaluronate OR hyaluronan OR emdogain OR "enamel matrix derivative*" OR "enamel matrix protein*" OR EMD | 21/06/2026 |
| Results | 264 |

4 Scopus

| Scopus | Data |
| --- | --- |
| TITLE-ABS-KEY ( ( autotransplant* OR "tooth transplant*" OR "teeth transplant*" OR "dental transplant*" OR "tooth autotransplant*" OR "teeth autotransplant*" OR "dental autotransplant*" OR "autogenous tooth transplant*" OR "autogenous teeth transplant*" OR "autogenous dental transplant*" OR "autologous tooth transplant*" OR "autologous teeth transplant*" OR "autologous dental transplant*" ) W/25 ( "enamel matrix derivative*" OR "enamel matrix protein*" OR emdogain OR EMD OR "platelet rich fibrin" OR "platelet-rich fibrin" OR PRF OR L-PRF OR A-PRF OR i-PRF OR "platelet rich plasma" OR "platelet-rich plasma" OR PRP OR PRGF OR "plasma rich in growth factors" OR "concentrated growth factor*" OR CGF OR "platelet lysate" OR "platelet concentrate*" OR "platelet derived growth factor" OR "platelet-derived growth factor" OR PDGF OR "PDGF-BB" OR rhPDGF OR "GEM 21S" OR becaplermin OR "fibroblast growth factor*" OR FGF OR "FGF-2" OR bFGF OR trafermin OR "bone morphogenetic protein*" OR BMP OR "BMP-2" OR "BMP-7" OR "transforming growth factor*" OR "TGF beta" OR TGF-beta OR "vascular endothelial growth factor" OR VEGF OR "insulin like growth factor" OR "insulin-like growth factor" OR IGF OR "hyaluronic acid" OR hyaluron* ) ) | 21/06/2026 |
| Results | 93 |

6. LILACS via the Virtual Health Library

| Coleção Lilcas Plus | Data |
| --- | --- |
| **Titulo, resumo, assunto:**  autotransplant* OR "auto-transplant*" OR "tooth autotransplant*" OR "teeth autotransplant*" OR "dental autotransplant*" OR "tooth transplant*" OR "teeth transplant*" OR "dental transplant*" OR "autogenous tooth transplant*" OR "autologous tooth transplant*" OR "autotransplante dentário" OR "autotransplante dental" OR "autotransplante de dente" OR "autotransplante de dentes" OR "transplante dentário autógeno" OR "transplante dentário autólogo" OR "transplante autógeno de dente" OR "transplante autólogo de dente" OR "trasplante dental autógeno" OR "trasplante dental autólogo" OR "autotrasplante dental" OR "autotrasplante dentario" OR "autotrasplante de diente" OR "autotrasplante de dientes"  **AND**  **Titulo, resumo, assunto:**  "enamel matrix derivative" OR "enamel matrix derivatives" OR "enamel matrix protein*" OR emdogain OR EMD OR "derivado da matriz do esmalte" OR "derivados da matriz do esmalte" OR "proteínas da matriz do esmalte" OR "derivado de la matriz del esmalte" OR "proteínas de la matriz del esmalte" OR "platelet-rich fibrin" OR "platelet rich fibrin" OR PRF OR L-PRF OR A-PRF OR i-PRF OR "fibrina rica em plaquetas" OR "fibrina rica en plaquetas" OR "platelet-rich plasma" OR "platelet rich plasma" OR PRP OR "plasma rico em plaquetas" OR "plasma rico en plaquetas" OR "plasma rich in growth factors" OR PRGF OR "plasma rico em fatores de crescimento" OR "plasma rico en factores de crecimiento" OR "concentrated growth factor*" OR CGF OR "fator de crescimento concentrado" OR "factores de crecimiento concentrados" OR "platelet concentrate*" OR "platelet lysate" OR "concentrado de plaquetas" OR "concentrado plaquetário" OR "concentrado plaquetario" OR "platelet-derived growth factor" OR PDGF OR PDGF-BB OR rhPDGF OR "fibroblast growth factor*" OR FGF OR FGF-2 OR bFGF OR trafermin OR "bone morphogenetic protein*" OR BMP OR BMP-2 OR BMP-7 OR "transforming growth factor beta" OR TGF-beta OR "TGF beta" OR VEGF OR "vascular endothelial growth factor" OR IGF OR "insulin-like growth factor" OR "hyaluronic acid" OR hyaluronan OR hyaluronate OR "ácido hialurônico" OR "ácido hialurónico" | 21/06/2026 |
| Results | 114 |

**ClinicalTrials.gov (complementary registry search)**

| ClinicalTrials | Data |
| --- | --- |
| Broad search: ("tooth autotransplantation" OR "dental autotransplantation" OR "autogenous tooth transplantation" OR "autologous tooth transplantation" OR "tooth transplantation") | 28/06/2026 |
| Results | 12 |
| Adjunct-specific search: ("tooth autotransplantation" OR "dental autotransplantation" OR "autogenous tooth transplantation" OR "autologous tooth transplantation" OR "tooth transplantation") AND ("platelet-rich fibrin" OR PRF OR "platelet-rich plasma" OR PRP OR "concentrated growth factor" OR CGF OR "enamel matrix derivative" OR EMD OR "plasma rich in growth factors" OR PRGF) |  |
| Results | 6 |

**Supplementary Tables**

**Supplementary Table S1. Reports excluded after full-text assessment and reasons for exclusion.**

| **Author / source, year** | **Title** | **Reason for exclusion** | **Exclusion category** |
| --- | --- | --- | --- |
| Moraes, 2018 | Transplante autógeno de terceiro molar inferior associado a I-PRF: relato de caso | Full text was unavailable despite retrieval attempts. | Full text not available |
| ClinicalTrials.gov | Clinical & Radiographic Evaluation of Concentrated Growth Factors (CGF) With Auto Transplanted Closed Apex Teeth (A Case Series Study) | Registry entry without published clinical outcome data. | Registry/protocol without results |
| ClinicalTrials.gov | Clinical Evaluation of Autogenous Tooth Transplantation With Instrumentally Simulated Open Apex and Autologous Platelet-Rich Fibrin (PRF) Support in Teeth With Complete Root Formation | Registry entry without published clinical outcome data. | Registry/protocol without results |
| Hamdani, 2025 | Autotransplantation of Immature Third Molars With Platelet-Rich Fibrin: A 5-Year Case Series | E-poster/clinical report presentation without full published clinical outcome data. | Conference abstract/presentation without sufficient clinical data |
| ClinicalTrials.gov | Auto Transplantation of Wisdom Teeth With or Without Platelet Rich Fibrin for Replacement of Non-Restorable Posterior Teeth | Registry entry without published clinical outcome data. | Registry/protocol without results |
| ClinicalTrials.gov | Efficacy of Digitally Tooth Autotransplantation With Adjunctive Use of Enamel Matrix Derivatives (EMD): A Prospective Case Series | Registry entry without published clinical outcome data. | Registry/protocol without results |
| ClinicalTrials.gov | Efficacy of Enamel Matrix Derivatives Adjunctive to Closed-apex Teeth Autotransplantation: A Randomized Clinical Trial | Registry entry without published clinical outcome data. | Registry/protocol without results |
| Calatrava, 2022 | Use of Adjunctive Enamel Matrix Derivatives Combined With Digitally Guided Tooth Autotransplantation: A Series of 10 Cases | Conference oral presentation/abstract without full published clinical outcome data. | Conference abstract/presentation without sufficient clinical data |
| Cobos, 2011 | Platelet-rich Plasma (PRP) as an Adjunct in the Healing of Dental Autotransplantation of Retained Lower Third Molar: A Case Report | Conference abstract without full published clinical data. | Conference abstract/presentation without sufficient clinical data |
| Fukuba, 2025 | The Apical Tooth Replantation With Surgical Intrusion Technique (ATR-SIT) for the Regenerative Treatment of Hopeless Teeth: A Report of Two Cases | Study focused on replantation/surgical intrusion rather than conventional tooth autotransplantation. | Ineligible procedure/model |
| Ali, 2017 | Autotransplantation of an Unerupted Wisdom Tooth Immediately After Removal of Grossly Destroyed Permanent Mandibular First Molar: A Case Report | Follow-up longer than 6 months was not clearly reported. | Follow-up ≤6 months or unclear |
| Cuesta Román, NR | Digitally Guided Modified Intentional Replantation for a Tooth With Hopeless Periodontal Prognosis: A Case Report | Study focused on intentional replantation rather than tooth autotransplantation. | Ineligible procedure/model |
| Anitua, 2026 | Case Report: Multidisciplinary Management With Dental Autotransplantation for Rehabilitation of a Post-traumatic Edentulous Maxillary Hemi-segment in a Growing Patient: Transverse Maxillary Development After 10 Years | The biological adjunct was not clearly applied to the tooth autotransplantation procedure; PRGF was used later for peri-implant/regenerative management. The report also represented an extended follow-up of a previously published case. | Adjunct not related to ATT/overlap |
| Singh, 2015 | Post-Odontoma Autotransplantation of an Impacted Tooth: A Case Report | Follow-up was limited to 6 months, which did not meet the predefined criterion of follow-up longer than 6 months. | Follow-up ≤6 months or unclear |
| Hoshino, 2000 | Application of Enamel Matrix Derivative for Tooth Transplantation and Replantation | Full text was not available in an eligible language. | Ineligible language |
| Falivene, 2025 | Autotransplantation of an Impacted Canine Using L-PRF: A Case Report | Full text was unavailable despite retrieval attempts. | Full text not available |
| Gjurčeski, 2024 | Use of Platelet-Enriched Fibrin in the Protocol for Autotransplantation of Teeth | Follow-up was limited to 3 months. | Follow-up ≤6 months or unclear |
| Sonmez, 2014 | Applications of Basic Fibroblastic Growth Factor (FGF-2, bFGF) in Dentistry | Review article; not an original clinical tooth autotransplantation study. | Review/non-original article |
| Easley-Perez, 2025 | The Use of Platelet-Rich Fibrin in Maintaining Vascularity for Intentionally Replanted Teeth: A Randomized Clinical Trial | Study focused on intentional replantation/replantation model rather than conventional tooth autotransplantation. | Ineligible procedure/model |
| Sha, 2025 | Application of Maxillary Sinus Lift Technique Combined With PRF in Immediate Autogenous Tooth Transplantation During Extraction | Full text was not available in an eligible language. | Ineligible language |
| Truong, 2020 | Dental Implant or Dental Transplant: A Two Case Report | Conference paper/abstract without sufficient full clinical outcome data. | Conference abstract/presentation without sufficient clinical data |
| Ninomiya, 2002 | Application of Enamel Matrix Derivative in Autotransplantation of an Impacted Maxillary Premolar: A Case Report | Follow-up was limited to 6 months, which did not meet the predefined criterion of follow-up longer than 6 months. | Follow-up ≤6 months or unclear |
| Premlata, 2014 | Immediate Autotransplantation of Immature Third Molar With Regeneration of Recipient Site Using Autologous Platelet-Rich Fibrin | Follow-up was limited to 6 months, which did not meet the predefined criterion of follow-up longer than 6 months. | Follow-up ≤6 months or unclear |

**Abbreviations:** ATT, autotransplantation; CGF, concentrated growth factor; EMD, enamel matrix derivative; I-PRF, injectable platelet-rich fibrin; L-PRF, leukocyte- and platelet-rich fibrin; NR, not reported; PRF, platelet-rich fibrin; PRGF, plasma rich in growth factors; PRP, platelet-rich plasma.

**Supplementary Table S2.** Detailed study characteristics and clinical categorization of included sources.

| **Study** | **Design / sample** | **Root maturity** | **Recipient-site status** | **Adjunct category** |
| --- | --- | --- | --- | --- |
| Hamamoto et al., 2005 | Case series; 2 patients / 2 teeth | Closed apex | Periodontal/root-surface defect | EMD |
| Marques-Ferreira et al., 2011 | Comparative clinical study; 26 patients / 28 teeth | Mixed | Mixed indications | EMD |
| Chaudhary et al., 2015 | Case report; 1 patient / 1 tooth | NR | Defective socket | PRF + graft |
| Miura et al., 2015 | Case report; 1 patient / 1 tooth | Closed apex | Alveolar cleft | PRP + graft |
| Ronchetti et al., 2015 | Retrospective analysis; 59 patients / 75 teeth | Mixed | Mixed posterior sites | EMD |
| Gonzalez-Ocasio and Stevens, 2017 | Case series; 10 patients / 11 teeth | Open apex | Fresh extraction socket | PRP |
| Alkofahi et al., 2020 | Case report; 1 patient / 1 tooth | Open apex | Fresh extraction socket | PRF |
| Gaviño-Orduña et al., 2020 | Case report; 1 patient / 1 tooth | Closed apex | Surgically prepared socket | PRGF |
| Mena-Álvarez et al., 2020 | Case report; 1 patient / 1 tooth | Closed apex | Digitally prepared socket | PRGF |
| Keranmu et al., 2021 | Comparative clinical study; 52 patients / 52 teeth | Closed apex | Infected/periapical lesion | CGF |
| Rey Lescure et al., 2021 | Case report; 1 patient / 2 teeth | Open apex | Fresh extraction socket | L-PRF |
| Anitua et al., 2022 | Case series; 2 patients / 3 teeth | Open apex | Trauma-related site | PRGF + graft |
| Sakhariya et al., 2023 | Case report; 1 patient / 1 tooth | Closed apex | Defective socket | PRF + PRP + graft |
| Alijani et al., 2023 | Case report; 1 patient / 1 tooth | Closed apex | Fresh extraction socket | PRF |
| Adamska et al., 2024 | Case report; 1 patient / 1 tooth | Open apex | Defective socket | A-PRF + graft |
| Pedrinaci et al., 2024 | Prospective case series; 12 patients / 13 teeth | Closed apex | Digitally prepared socket | EMD |
| Shetty et al., 2024 | Case report; 2 patients / 2 teeth | NR; managed as mature teeth with intentional RCT | Surgically prepared socket | PRF |
| Ainiwaer et al., 2024 | Retrospective case series; 36 patients / 36 teeth | Closed apex | Defective socket | CGF + graft |
| Ainiwaer and Wang, 2025 | Case report; 1 patient / 2 teeth | Closed apex | Infected/periapical lesion | CGF |
| Genc et al., 2025 | Comparative clinical study; 20 patients / 20 teeth | Mixed | Mixed molar sites | PRP |
| SamavatiJame et al., 2025 | Case report; 2 patients / 2 teeth | Open apex | Fresh extraction socket | L-PRF |
| Ma et al., 2026 | Case report; 1 patient / 1 tooth | Open apex | Digitally prepared socket | PRF |

**Abbreviations:** A-PRF, advanced platelet-rich fibrin; CGF, concentrated growth factor; EMD, enamel matrix derivative; L-PRF, leukocyte- and platelet-rich fibrin; NR, not reported; PRF, platelet-rich fibrin; PRGF, plasma rich in growth factors; PRP, platelet-rich plasma; RCT, root canal treatment.

**Supplementary Table S3.** Detailed biological adjuncts and procedural protocols of included sources.

| **Study** | **Adjunct protocol / application** | **Co-interventions** | **Stabilization** | **Endodontic approach** | **Pulp/sensibility assessment** |
| --- | --- | --- | --- | --- | --- |
| Hamamoto et al., 2005 | EMD applied to the entire root surface after mechanical debridement | Root-surface debridement | Wire-and-resin splint for 1 month; wire diameter NR | RCT started 3 weeks after transplantation in one case; canal obliteration limited treatment in the second case | NR |
| Marques-Ferreira et al., 2011 | EMD or saline applied topically to the root surface before transplantation | Immediate or early-healing recipient socket protocols | Cross-occlusal silk sutures for 10 days | Selective RCT when signs of necrosis or periapical pathology were detected | Cold test and percussion test |
| Chaudhary et al., 2015 | PRF gel and PRF membrane placed in the recipient defect and around the transplanted tooth | DFDBA | Periodontal wire splint reinforced with Erich arch bar; removed after 4 weeks; wire diameter NR | RCT on postoperative day 10 | NR |
| Miura et al., 2015 | PRP mixed with autogenous cancellous bone and marrow and packed into the alveolar cleft before transplantation | Iliac PCBM graft | Orthodontic bracket tied to main archwire; duration NR | NR | NR |
| Ronchetti et al., 2015 | EMD use recorded as present or absent | NR | NR | RCT recorded when required; not considered failure | NR |
| Gonzalez-Ocasio and Stevens, 2017 | PRP injected into the recipient socket before final tooth placement | NR | Orthodontic wire and composite splint for 1 month; wire diameter NR | No RCT required | Cold test |
| Alkofahi et al., 2020 | PRF clot placed in the recipient socket before donor tooth placement | NR | Rigid splinting; duration and wire diameter NR | No RCT | Vital pulp testing reported |
| Gaviño-Orduña et al., 2020 | PRGF injected into the apical third; activated PRGF clot used to fill the apical area | Digital planning, 3D tooth replica, and fragile fracture apicoectomy | 4-0 silk suture; no wire splint | No RCT | Cold test |
| Mena-Álvarez et al., 2020 | Tooth stored in PRGF during extraoral phase; activated PRGF applied around the tooth after placement | Fully digital workflow and surgical guide | Cross-occlusal suture splint for 7 days | RCT at 2 weeks | NR |
| Keranmu et al., 2021 | CGF membrane placed in the socket to cover the inflammatory area and support the transplanted root | Curettage and saline irrigation of infected sites | Figure-of-eight suture for 1 week when stability was good; fiberglass band for 4 weeks when stability was poor | RCT in all teeth at 2–4 weeks | NR |
| Rey Lescure et al., 2021 | L-PRF membranes placed in the recipient sites; donor teeth stored in L-PRF exudate | NR | Figure-of-eight cross suture for 1 week | No RCT | Vital pulp testing reported |
| Anitua et al., 2022 | PRGF used to irrigate the socket, prepare sticky bone, and cover the surgical area with fibrin membranes | Autologous bone graft | Figure-of-eight suture or splinting; duration NR | No RCT | Pulp vitality reported; test method NR |
| Sakhariya et al., 2023 | PRF placed at the apex and socket walls; PRP mixed with autogenous bone chips to fill the socket | Autogenous interradicular bone chips | 24G stainless-steel wire splint; duration not clearly reported | Extraoral RCT before transplantation | Not applicable / NR |
| Alijani et al., 2023 | PRF membrane applied to socket walls; PRF exudate used with saline irrigation | None reported | Simple suture and ligature wire splint for 2 weeks; wire diameter NR | RCT before surgery | NR |
| Adamska et al., 2024 | A-PRF mixed with autogenous dentin graft as “sticky tooth”; A-PRF membrane placed over the graft | Autogenous dentin graft | Sectional 0.016-inch stainless-steel wire for 3 months | No initial RCT | Cold test |
| Pedrinaci et al., 2024 | EMD applied directly to the donor root surface before placement | Digital planning, surgical guide, and tooth replica | Semi-rigid orthodontic wire splint for 2–4 weeks; wire diameter NR | Prophylactic RCT in all teeth at 2–4 weeks | Not applicable / NR |
| Shetty et al., 2024 | PRF inserted into the recipient socket before transplantation to cover the root surface | Physio-dispenser socket preparation; allogenic bone graft and bioresorbable membrane in one case | Non-rigid splinting for 2 weeks using archwire or twisted ligature wire; wire diameter NR | Intentional RCT initiated after 1 week, with calcium hydroxide for 2 weeks followed by obturation | Not applicable after RCT |
| Ainiwaer et al., 2024 | CGF membranes placed over grafted recipient defects and crestal area | Artificial bone powder | Figure-of-eight suture for 1 week or fiberglass band for 4 weeks, according to initial stability | RCT in all mature teeth, usually 2–4 weeks after transplantation | NR |
| Ainiwaer and Wang, 2025 | CGF membranes placed in the recipient sockets to fill periapical defects before donor tooth placement | Digital tooth replicas | Figure-of-eight cross suture for 7 days | RCT reported; timing not clearly described | NR |
| Genc et al., 2025 | PRP applied to the recipient site in the PRP groups | NR | Horizontal figure-of-eight mattress suture with 3-0 silk for 1 week; no additional wire splint reported | No routine prophylactic RCT; RCT only for non-vital teeth with pathological changes | Thermal vitality test |
| SamavatiJame et al., 2025 | L-PRF membranes packed into the socket and wrapped around the transplanted tooth | NR | Suture-supported stabilization with non-resorbable sutures; no wire splint reported | No RCT | Vitality inferred from continued root development and absence of pathology; test method NR |
| Ma et al., 2026 | PRF applied to the recipient site; donor tooth immersed in PRF; donor socket filled with PRF | Orthodontic donor activation, digital planning, 3D-printed donor replica, osteotomy guide, and seating guide | Interrupted sutures and twisted-wire/resin splint for 2 months; wire diameter NR | No prophylactic RCT | Electric pulp test |

**Abbreviations:** A-PRF, advanced platelet-rich fibrin; CGF, concentrated growth factor; DFDBA, demineralized freeze-dried bone allograft; EMD, enamel matrix derivative; L-PRF, leukocyte- and platelet-rich fibrin; NR, not reported; PCBM, particulate cancellous bone and marrow; PRF, platelet-rich fibrin; PRGF, plasma rich in growth factors; PRP, platelet-rich plasma; RCT, root canal treatment.

**Supplementary Table S4.** Adjunct preparation protocols, centrifugation parameters, and application sites.

| **Study** | **Adjunct** | **Preparation protocol** | **Centrifugation parameters** | **Application location / timing** |
| --- | --- | --- | --- | --- |
| Hamamoto et al., 2005 | EMD | EMD applied after mechanical debridement of the denuded root surface | Not applicable | Entire donor root surface before transplantation |
| Marques-Ferreira et al., 2011 | EMD | EMD applied topically to the donor root surface; saline used in control sites | Not applicable | Donor root surface before transplantation |
| Chaudhary et al., 2015 | PRF + DFDBA | Venous blood processed to obtain PRF; PRF clot mixed with DFDBA to form PRF gel/allograft mixture | 3000 rpm, approximately 400 g, 10 min | Recipient bony defect; PRF membrane also placed over/around the transplanted tooth |
| Miura et al., 2015 | PRP + PCBM | PRP prepared from venous blood and mixed with autogenous particulate cancellous bone and marrow | Not reported | Alveolar cleft/recipient defect before tooth transplantation |
| Ronchetti et al., 2015 | Enamel matrix proteins / EMD | Use recorded as present or absent | Not applicable | Application protocol not reported |
| González-Ocasio and Stevens, 2017 | PRP | PRP prepared from venous blood and injected after donor tooth adaptation | Not reported | Recipient socket before final donor tooth placement |
| Alkofahi et al., 2020 | PRF | PRF clot prepared from venous blood | 3000 rpm, 10 min | Recipient socket before donor tooth placement; no root-surface application |
| Gaviño-Orduña et al., 2020 | PRGF | PRGF prepared from venous blood; non-activated PRGF and activated PRGF fibrin clot used | 580 g, 8 min | Non-activated PRGF injected into apical third; activated PRGF clot used to fill/stabilize apical area |
| Mena-Álvarez et al., 2020 | PRGF | Donor tooth stored in PRGF during extraoral phase; activated PRGF applied after placement | Not reported | Extraoral storage medium and around the transplanted tooth after placement |
| Keranmu et al., 2021 | CGF | CGF prepared from autologous blood; clot compressed into membrane | Medifuge protocol; detailed rpm/time not reported | Bottom of recipient socket, wrapping root and covering inflammatory area |
| Rey Lescure et al., 2021 | L-PRF | Peripheral blood collected in 9-mL tubes; clots transferred to Xpression box to obtain membranes | 2700 rpm, 12 min | Recipient sockets; donor teeth stored in L-PRF exudate |
| Anitua et al., 2022 | PRGF + autogenous bone | PRGF fractions used to irrigate socket, store bone chips, prepare sticky bone, and form fibrin membranes | 580 g, 8 min | Socket irrigation, bone-chip preparation, labial graft area, and coverage with PRGF membranes |
| Alijani et al., 2023 | PRF membrane/exudate | PRF clot compressed in PRF box to form membrane | 2000 rpm, 400 g, 10 min | Socket walls; recipient socket also irrigated with PRF exudate and saline |
| Sakhariya et al., 2023 | PRF + PRP + autogenous bone chips | PRF prepared and placed in socket; bone chips mixed with PRP | PRF: 3000 rpm, 10 min; PRP: not reported | PRF placed at apex and socket walls; PRP-bone mixture used to fill socket before donor placement |
| Adamska et al., 2024 | A-PRF + autogenous dentin graft | A-PRF prepared from venous blood; autogenous dentin graft mixed with A-PRF as “sticky tooth” | 1500 rpm, 14 min | Socket/defect filling with A-PRF–dentin graft; A-PRF membrane placed over graft |
| Pedrinaci et al., 2024 | EMD | EMD gel applied directly after atraumatic extraction and guided bed preparation | Not applicable | Donor root surface before placement into prepared socket |
| Shetty et al., 2024 | PRF membrane | PRF clot prepared from venous blood collected without anticoagulant | 3000 rpm, 10 min | Recipient socket before transplantation, aiming to cover the root with PRF scaffold |
| Ainiwaer et al., 2024 | CGF + artificial bone powder | Bio-Oss packed around root/defects; CGF membrane pressed and placed over graft/crest | Not reported | Around root, bone defects, and crest after transplantation |
| Ainiwaer and Wang, 2025 | CGF | CGF prepared from venous blood in silica-coated tube; CGF layer separated with sterile scissors | 30 s acceleration; 2 min at 2700 rpm; 4 min at 2400 rpm; 4 min at 2700 rpm; 3 min at 3000 rpm; 36 s deceleration | Recipient sockets/periapical defects before donor tooth placement; no root-surface application |
| Genc et al., 2025 | PRP | PRP prepared from venous blood using two-step centrifugation | 3500 rpm, 12 min; then 3000 rpm, 15 min | Recipient site during surgery |
| SamavatiJame et al., 2025 | L-PRF / PRF | L-PRF membranes prepared from venous blood | Approximately 400 g, 12 min | Two to three membranes packed into socket; one membrane wrapped around transplanted tooth before closure |
| Ma et al., 2026 | PRF | PRF prepared from venous blood; detailed protocol referred to appendix in original report | Not reported in main text | Recipient site; donor tooth immersed in PRF; donor socket filled with PRF |

**Abbreviations:** A-PRF, advanced platelet-rich fibrin; CGF, concentrated growth factor; DFDBA, demineralized freeze-dried bone allograft; EMD, enamel matrix derivative; L-PRF, leukocyte- and platelet-rich fibrin; PCBM, particulate cancellous bone and marrow; PRF, platelet-rich fibrin; PRGF, plasma rich in growth factors; PRP, platelet-rich plasma.

**Supplementary Table S5.** Detailed follow-up and reported outcomes of included sources.

| **Study** | **Follow-up** | **Main outcomes reported** | **Complications/failures** | **Overall finding** |
| --- | --- | --- | --- | --- |
| Hamamoto et al., 2005 | 26–30 months | Probing attachment level, probing depth, mobility, percussion, prosthetic function | No pain, abnormal mobility, or progressive periodontitis reported | Both transplanted molars healed uneventfully and functioned as prosthetic abutments |
| Marques-Ferreira et al., 2011 | 24–65 months | Survival/success, pulp status, root canal need, periodontal status, root development/canal obliteration | Two teeth lost due to persistent apical periodontitis | Overall favorable prognosis; pulp outcomes were better in open-apex teeth, with no clear difference between EMD and saline |
| Chaudhary et al., 2015 | 12 months | Clinical healing, bone loss, root resorption, ankylosis | No bone loss, root resorption, or ankylosis reported | Uneventful healing after ATT with PRF and DFDBA in a large bony defect |
| Miura et al., 2015 | 27 months | Tooth stability, gingival contour, bone formation, periodontal space, lamina dura, root resorption | No infection or root resorption reported | Stable transplanted tooth with new bone formation in the alveolar cleft |
| Ronchetti et al., 2015 | 0.44–12.28 years | Success, tooth type, root development, EMD use, surgeon effect | Lower success in molars than premolars; failures recorded in long-term follow-up | Tooth type and surgeon were associated with success; EMD was not significantly associated with outcome |
| Gonzalez-Ocasio and Stevens, 2017 | 12 months | Tooth function, mobility, probing depth, pulp vitality, root development, RCT need | No necrosis, infection, external root resorption, or failures reported | All transplanted immature third molars remained functional, vital, and showed continued root development |
| Alkofahi et al., 2020 | 36 months | Symptoms, mobility, occlusion, pulp vitality, root development, root resorption, ankylosis, periapical pathology | No root resorption, ankylosis, or periapical pathology reported | Immature third molar remained asymptomatic and showed continued root development |
| Gaviño-Orduña et al., 2020 | 36 months | Symptoms, pulp sensibility, canal obliteration, occlusion, root resorption, periapical status | No inflammatory or replacement resorption and no periapical radiolucency reported | Mature premolar remained functional and responsive to pulp testing after PRGF-assisted ATT |
| Mena-Álvarez et al., 2020 | 24 months | Symptoms, function, marginal periodontal status, bone regeneration, PDL space, root resorption, ankylosis | No postoperative complications, root resorption, or ankylosis reported | Functional transplanted tooth with normal PDL space and uneventful healing |
| Keranmu et al., 2021 | Mean 26 months | Success, periapical bone healing, inflammation resolution, pain, initial stability, mobility, periodontal status, resorption, ankylosis | No resorption or ankylosis in surviving teeth; failures not prominent | CGF group showed faster early healing and better initial stability, but interpretation is limited by study design |
| Rey Lescure et al., 2021 | 24 months | Pulp vitality, mobility, apical closure, infection, periapical radiolucency, root resorption | No infection, periapical radiolucency, or root resorption reported | Both immature third molars remained vital and showed apical closure |
| Anitua et al., 2022 | 3–7 years | Vitality, root development, apex closure, symptoms, periodontal health, aesthetics, resorption, apical pathology | No loss of vitality, resorption, or apical pathology reported | Immature transplanted teeth remained vital and functional with complete apex closure |
| Sakhariya et al., 2023 | 37 months | Periodontal stability, mobility, ankylosis, periapical pathology, bone loss, function | Minimal mesial bone loss and mild bleeding on probing reported | Transplanted tooth remained functional and periodontally stable after combined PRF, PRP, and bone chip use |
| Alijani et al., 2023 | 4 years | Symptoms, ankylosis, replacement resorption, inflammation, periapical pathology, lamina dura | No ankylosis, resorption, inflammation, or periapical pathology reported | Stable and functional outcome after PRF-assisted ATT |
| Adamska et al., 2024 | 12 months | Soft-tissue healing, mobility, probing depth, pulp status, root development, bone defect reconstruction, resorption, periapical status | Pulp necrosis, inflammatory root resorption, increased mobility, persistent radiolucency, and transplant extraction | Bone defect reconstruction was achieved, but the transplanted tooth failed |
| Pedrinaci et al., 2024 | 24 months | Survival, success, periodontal parameters, clinical attachment, ridge dimensions, root resorption, function, patient satisfaction | No progressive inflammatory or replacement root resorption reported | High survival and success after EMD-assisted ATT with digital workflow; no control group |
| Shetty et al., 2024 | 24 months | Symptoms, function, mobility, inflammation, PDL space, ankylosis, bone growth | No pathological mobility, inflammation, ankylosis, or radiographic failure reported | Both transplanted canines remained asymptomatic and functional, with patent PDL space |
| Ainiwaer et al., 2024 | Mean 42.7 months | Success, probing depth, mobility, symptoms, bone density, function, root resorption, bone loss | Three failures due to root resorption or bone loss | Most mature third molars remained functional, with 91.7% success at mid-term follow-up |
| Ainiwaer and Wang, 2025 | 16–18 months | Symptoms, function, mobility, occlusion, periapical healing, PDL space, lamina dura, ankylosis, resorption | No ankylosis, internal/external resorption, or recurrent pathology reported | Bilateral mature third molars showed stable clinical and radiographic healing |
| Genc et al., 2025 | 12 months | Pulp vitality, probing depth, mobility, pain, root length, root resorption | Inflammatory resorption in 11/20 teeth, replacement resorption in 5/20, and two failures | PRP did not significantly improve short-term outcomes compared with controls |
| SamavatiJame et al., 2025 | 24 and 84 months | Symptoms, function, stability, root development, ankylosis, radiographic pathology, RCT need | No ankylosis or radiographic pathology reported | Immature third molars showed continued root development and no need for RCT |
| Ma et al., 2026 | 24 months | Function, pulp sensibility, apical closure, alveolar bone stability, donor-site bone regeneration, root resorption, ankylosis | No external root resorption or ankylosis reported | Immature impacted third molar remained functional, responsive to EPT, and showed near-complete apical closure |

**Abbreviations:** ATT, autotransplantation; CGF, concentrated growth factor; DFDBA, demineralized freeze-dried bone allograft; EMD, enamel matrix derivative; EPT, electric pulp test; PDL, periodontal ligament; PRF, platelet-rich fibrin; PRGF, plasma rich in growth factors; PRP, platelet-rich plasma; RCT, root canal treatment.

**Supplementary Table S6.** JBI Critical Appraisal Checklist for Case Reports.

| **Study** | **Q1**  **Demographics** | **Q2**  **History/timeline** | **Q3**  **Clinical condition** | **Q4**  **Diagnostic tests/assessment** | **Q5 Intervention/procedure** | **Q6**  **Post-intervention condition** | **Q7**  **Adverse events/harms** | **Q8 Takeaway lesson** | **Overall appraisal** |
| --- | --- | --- | --- | --- | --- | --- | --- | --- | --- |
| Chaudhary et al., 2015 | Y | Y | Y | Y | Y | Y | Y | Y | Include |
| Miura et al., 2015 | Y | Y | Y | Y | Y | Y | Y | Y | Include |
| Alkofahi et al., 2020 | Y | Y | Y | Y | Y | Y | Y | Y | Include |
| Gaviño-Orduña et al., 2020 | Y | Y | Y | Y | Y | Y | Y | Y | Include |
| Mena-Álvarez et al., 2020 | Y | Y | Y | Y | Y | Y | Y | Y | Include |
| Rey Lescure et al., 2021 | Y | Y | Y | Y | Y | Y | Y | Y | Include |
| Sakhariya et al., 2023 | Y | Y | Y | Y | Y | Y | Y | Y | Include |
| Alijani et al., 2023 | Y | Y | Y | Y | Y | Y | Y | Y | Include |
| Adamska et al., 2024 | Y | Y | Y | Y | Y | Y | Y | Y | Include |
| Ainiwaer and Wang, 2025 | Y | Y | Y | Y | Y | Y | Y | Y | Include |
| SamavatiJame et al., 2025 | Y | Y | Y | Y | Y | Y | Y | Y | Include |
| Ma et al., 2026 | Y | Y | Y | Y | Y | Y | Y | Y | Include |
| Shetty et al., 2024 | Y | Y | Y | Y | Y | Y | Y | Y | Include |

Notes: Q1, patient demographic characteristics clearly described; Q2, patient history clearly described and presented as a timeline; Q3, current clinical condition clearly described; Q4, diagnostic tests or assessment methods and results clearly described; Q5, intervention or treatment procedure clearly described; Q6, post-intervention clinical condition clearly described; Q7, adverse events or unanticipated events identified and described; Q8, takeaway lessons provided.

Abbreviations: JBI, Joanna Briggs Institute; Y, yes.

**Supplementary Table S7.** JBI Critical Appraisal Checklist for Case Series.

| **Study** | **Q1**  **Clear inclusion criteria** | **Q2 Standard/reliable measurement** | **Q3**  **Valid identification methods** | **Q4 Consecutive inclusion** | **Q5 Complete inclusion** | **Q6 Demographics reported** | **Q7**  **Clinical information reported** | **Q8 Outcomes/follow-up reported** | **Q9 Site/clinic information** | **Q10 Appropriate statistical analysis** | **Overall appraisal** |
| --- | --- | --- | --- | --- | --- | --- | --- | --- | --- | --- | --- |
| Hamamoto et al., 2005 | U | Y | Y | U | U | Y | Y | Y | U | NA | Include with caution |
| Gonzalez-Ocasio and Stevens, 2017 | Y | Y | Y | U | U | U | Y | Y | Y | Y | Include |
| Anitua et al., 2022 | U | Y | Y | U | U | Y | Y | Y | Y | NA | Include with caution |
| Pedrinaci et al., 2024 | Y | Y | Y | U | U | Y | Y | Y | Y | Y | Include |
| Ainiwaer et al., 2024 | Y | Y | Y | U | U | Y | Y | Y | Y | Y | Include |

Notes: Q1, clear criteria for inclusion in the case series; Q2, standard and reliable measurement of the condition; Q3, valid methods used for identifying the condition; Q4, consecutive inclusion of participants; Q5, complete inclusion of participants; Q6, clear reporting of participant demographics; Q7, clear reporting of clinical information; Q8, clear reporting of outcomes or follow-up results; Q9, clear reporting of presenting site or clinic information; Q10, appropriate statistical analysis.

Abbreviations: JBI, Joanna Briggs Institute; NA, not applicable; U, unclear; Y, yes.

**Supplementary Table S8.** ROBINS-I assessment for comparative and retrospective studies.

| **Study** | **D1 Confounding** | **D2**  **Selection of participants** | **D3 Classification of interventions** | **D4**  **Deviations from intended interventions** | **D5**  **Missing data** | **D6**  **Measurement of outcomes** | **D7**  **Selection of reported result** | **Overall risk of bias** |
| --- | --- | --- | --- | --- | --- | --- | --- | --- |
| Marques-Ferreira et al., 2011 | Serious | Moderate | Low | Moderate | Moderate | Moderate | Moderate | Serious |
| Ronchetti et al., 2015 | Serious | Low | Moderate | Moderate | Moderate | Moderate | Moderate | Serious |
| Keranmu et al., 2021 | Serious | Moderate | Low | Moderate | Moderate | Moderate | Moderate | Serious |
| Genc et al., 2025 | Moderate | Moderate | Low | Moderate | Serious | Moderate | Moderate | Serious |

Notes: D1, bias due to confounding; D2, bias in selection of participants into the study; D3, bias in classification of interventions; D4, bias due to deviations from intended interventions; D5, bias due to missing data; D6, bias in measurement of outcomes; D7, bias in selection of the reported result.

Abbreviations: NI, no information; ROBINS-I, Risk Of Bias In Non-randomized Studies of Interventions.

**Supplementary Figure S1.** JBI critical appraisal of case reports. The figure summarizes domain-level judgments for the included case reports. Q1, patient demographic characteristics clearly described; Q2, patient history clearly described and presented as a timeline; Q3, current clinical condition clearly described; Q4, diagnostic tests or assessment methods and results clearly described; Q5, intervention or treatment procedure clearly described; Q6, post-intervention clinical condition clearly described; Q7, adverse events or unanticipated events identified and described; Q8, takeaway lessons provided. Green indicates yes. **Abbreviations**: JBI, Joanna Briggs Institute
